# In-Depth Characterization of the *Clostridioides difficile* Phosphoproteome to Identify Ser/Thr Kinase Substrates

**DOI:** 10.1016/j.mcpro.2022.100428

**Published:** 2022-10-14

**Authors:** Transito Garcia-Garcia, Thibaut Douché, Quentin Giai Gianetto, Sandrine Poncet, Nesrine El Omrani, Wiep Klaas Smits, Elodie Cuenot, Mariette Matondo, Isabelle Martin-Verstraete

**Affiliations:** 1 Laboratoire Pathogénese des Bactéries Anaérobies, UMR CNRS 6047, Institut Pasteur, Université Paris Cité, Paris, France; 2 Plateforme Protéomique, Unité de Technologie et Service Spectrométrie de Masse pour la biologie, CNRS USR 2000, Institut Pasteur, Université Paris Cité, Paris, France; 3 Hub de bioinformatique et biostatistiques, Departement de Biologie computationelle, Institut Pasteur, Université Paris Cité, Paris, France; 4 INRAE, AgroParisTech, Micalis Institute, Université Paris-Saclay, Jouy-en-Josas, France; 5 Department of Medical Microbiology, Leiden University Medical Center, Leiden, The Netherlands; 6 Institut Universitaire de France, Paris, France

**Keywords:** Hanks kinase, phosphatase, serine phosphorylation, threonine phosphorylation, cell division, envelope homeostasis, ABC, ammonium bicarbonate, ACN, acetonitrile, AGC, automatic gain control, ATc, anhydrotetracycline, EB, exchange buffer, FA, formic acid, FDR, false discovery rate, HA, hemagglutinin, HCD, higher energy collisional dissociation, HFBA, heptafluorobutyric acid, KD, kinase domain, LP, localization probability, MS, mass spectrometry, PASTA, penicillin-binding and STK-associated, PG, peptidoglycan, pS, phosphoserine, pT, phosphothreonine, PTM, post-translational modification, PTS, phosphotransferase system, S, serine, STK, serine/threonine kinase, STP, serine/threonine phosphatase, T, threonine, TiO_2_, titanium dioxide, Y, tyrosine

## Abstract

*Clostridioides difficile* is the leading cause of postantibiotic diarrhea in adults. During infection, the bacterium must rapidly adapt to the host environment by using survival strategies. Protein phosphorylation is a reversible post-translational modification employed ubiquitously for signal transduction and cellular regulation. Hanks-type serine/threonine kinases (STKs) and serine/threonine phosphatases have emerged as important players in bacterial cell signaling and pathogenicity. *C. difficile* encodes two STKs (PrkC and CD2148) and one phosphatase. We optimized a titanium dioxide phosphopeptide enrichment approach to determine the phosphoproteome of *C. difficile*. We identified and quantified 2500 proteins representing 63% of the theoretical proteome. To identify STK and serine/threonine phosphatase targets, we then performed comparative large-scale phosphoproteomics of the WT strain and isogenic ΔprkC, CD2148, Δstp, and prkC CD2148 mutants. We detected 635 proteins containing phosphorylated peptides. We showed that PrkC is phosphorylated on multiple sites in vivo and autophosphorylates *in vitro*. We were unable to detect a phosphorylation for CD2148 *in vivo*, whereas this kinase was phosphorylated *in vitro* only in the presence of PrkC. Forty-one phosphoproteins were identified as phosphorylated under the control of CD2148, whereas 114 proteins were phosphorylated under the control of PrkC including 27 phosphoproteins more phosphorylated in the ∆stp mutant. We also observed enrichment for phosphothreonine among the phosphopeptides more phosphorylated in the Δstp mutant. Both kinases targeted pathways required for metabolism, translation, and stress response, whereas cell division and peptidoglycan metabolism were more specifically controlled by PrkC-dependent phosphorylation in agreement with the phenotypes of the ΔprkC mutant. Using a combination of approaches, we confirmed that FtsK was phosphorylated *in vivo* under the control of PrkC and that Spo0A was a substrate of PrkC *in vitro*. This study provides a detailed mapping of kinase–substrate relationships in *C. difficile*, paving the way for the identification of new biomarkers and therapeutic targets.

## Highlights

• An optimized phosphopeptide enrichment protocol for *Clostridioides difficile*.
• About 117 phosphorylation events under the control of the PASTA kinase, PrkC.
• About 27 PrkC-dependent targets were more phosphorylated in a phosphatase mutant.
• About 41 phosphorylation events under the control of the second Hanks-kinase CD2148.

## Graphical Abstract

**Figure:**
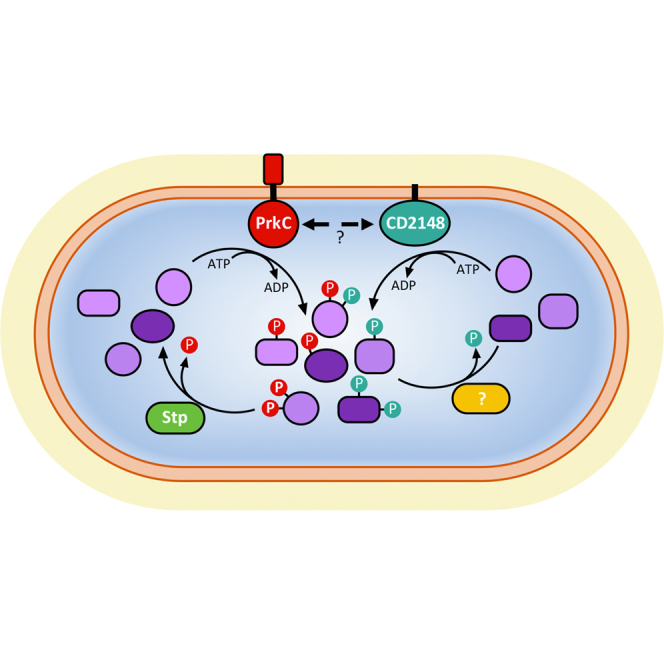

## In Brief

An optimized workflow for phosphopeptide enrichment has been developed in *Clostridioides difficile* revealing extensive protein phosphorylation. Using kinase or phosphatase mutant strains and quantitative proteomics, 41 and 114 downstream targets of the kinases PrkC and CD2148, respectively, were identified in *C. difficile*. We confirmed the phosphorylation of PrkC and Spo0A *in vitro* and of FtsK *in vivo*.

The survival of an organism depends on its capacity to quickly respond and adapt to constantly changing environmental conditions. Protein phosphorylation is a reversible post-translational modification (PTM) employed not only in eukaryotes but also in prokaryotes for signal transduction and cellular regulation (1, 2). In bacteria, phosphorylation has initially been thought to occur strictly on histidine and aspartate residues mediated by the histidine kinases of two-component systems (3). Albeit less abundant than in eukaryotes, data are increasing that show the existence of phosphorylation at serine (S), threonine (T), and tyrosine (Y) and demonstrate their crucial role in bacteria. Similar to eukaryotes, phosphorylations on S and T are mediated by Hanks-type serine/threonine kinases (STKs), whereas specific serine/threonine phosphatases (STPs) are involved in their dephosphorylation (4, 5). STKs phosphorylate a broad spectrum of substrates controlling many cellular processes, such as cell division, cell wall biosynthesis, central carbon metabolism, sporulation, biofilm formation, virulence, and host–pathogen interactions (6, 7, 8). Over the last decade, S/T/Y phosphorylation events have been detected using *in vitro* kinase assays and phosphoproteomic approaches in both Gram-negative and Gram-positive bacteria, including important pathogens (9, 10). Mass spectrometry (MS)–based techniques have become the method of choice for studying PTMs including phosphorylation. The substoichiometric nature of protein phosphorylation, combined with a lower level of phosphorylation at S/T/Y residues in bacteria compared with eukaryotes, has long made bacterial quantitative phosphoproteomics difficult. Several large-scale phosphoproteome analyses using large amounts of proteins have been reported in bacteria, including *Escherichia coli* (11), *Bacillus subtilis* (12, 13), *Streptococcus pneumoniae* (14), *Acinetobacter baumanii* (15, 16), and *Streptococcus thermophilus* (17). Recent improvements in phosphopeptide enrichment, liquid chromatography, and MS have increased the sensitivity of detection of phosphosites in bacteria (18, 19, 20). The phosphorylated proteins identified by phosphoproteomic studies are rather diverse among the tested firmicutes and, in general, a low overlap for STK targets is reported (18, 19). For Clostridia, a first study has identified 61 phosphorylated proteins in *Clostridium acetobutylicum* (21), whereas very recently, a large-scale phosphoproteome of *Clostridioides difficile* has been obtained (22). However, to date, the role of STK and STP on global phosphorylation has not been analyzed for any clostridial species.

*C. difficile* is a Gram-positive, spore-forming, anaerobic bacterium and is the leading cause of antibiotic-associated nosocomial diarrhea (23). *C. difficile* infection is a challenging threat to human health (24, 25). New strategies to effectively treat *C. difficile* infection are urgently needed. Thus, a better understanding of molecular basis of important components for bacterial survival and pathogenesis regulation is of crucial importance. The role of PTMs by phosphorylation in the control of key steps of the infectious cycle remains poorly understood in this major enteropathogen. The *C. difficile* genome encodes two Hanks-type STKs, PrkC (CD2578) and CD2148, and one STP, CD2579. The *prkC* and *stp* genes are cotranscribed, whereas *CD2148* is monocistronic. PrkC is a transmembrane kinase with a cytosolic N-terminal Hanks-type kinase domain (KD) and a C-terminal extracellular signal receptor domain containing two penicillin-binding and STK-associated (PASTA) repeats (6). We previously demonstrated that PrkC is localized at the division site and involved in the control of cell division, cell wall metabolism, and antibiotic resistance (26). However, CwlA, which is an endopeptidase-hydrolyzing peptidoglycan (PG), is the sole PrkC substrate identified so far. The PrkC-dependent phosphorylation of CwlA controls its localization at the cell wall and modulates cell division (27). CD2148 is a kinase lacking extracellular PASTA domains that is anchored in the cell wall and enriched at the septum of the cells. CD2148 is involved in cell separation by a CwlA-dependent mechanism. CD2148 modulates the level of PrkC-dependent phosphorylation of CwlA and its exposure at the cell surface through an unknown molecular mechanism (27).

In this work, we used mutants inactivated for genes encoding PrkC, CD2148, or STP to establish a comparative phosphoproteomic analysis in order to identify their respective candidate targets. Phosphoproteomic analysis showed extensive changes in protein phosphorylation in the Δ*prkC* mutant and comparatively fewer changes in the *CD2148* mutant. Our results identify numerous proteins of the cell wall, cell division complex, as well as several metabolic proteins, as potential targets of the *C. difficile* STKs. Our findings provide a better understanding of potential kinase–substrate relationships and a valuable resource for targeted investigation of mechanisms by which protein phosphorylation regulates pathways required for growth and pathogenesis in *C. difficile*.

## Experimental Procedures

### Bacterial Strains, Growth Conditions, and Phenotypic Tests

*C. difficile* strains were routinely grown at 37 °C in an anaerobic environment (90% N_2_, 5% CO_2_, and 5% H_2_) in TY (Bacto tryptone 30 g/l, yeast extract 20 g/l, pH 7.4) or in BHI (Difco). When necessary, *C. difficile* culture media were supplemented with cefoxitin (25 μg/ml), thiamphenicol (7.5 μg/ml), and erythromycin (2.5 μg/ml). *E. coli* strains were cultured at 37 °C in LB broth (Bacto tryptone 10 g/l, yeast extract 5 g/l, and NaCl 0.5 g/l), containing chloramphenicol (15 μg/ml) or ampicillin (100 μg/ml). Anhydrotetracycline (ATc) was used to induce the expression of the *ftsK* gene from the P_*tet*_-inducible promoter of pDIA6103 (28). Bacterial strains and plasmids used in this study are listed in supplemental Table S1. Exponential growing *C. difficile* cells (BHI) were exposed to 50 °C for 15 min. After serial dilution, the samples without or with a heat treatment were plated on BHI agar plates and incubated for 24 h at 37 °C. Overnight cultures of *C. difficile* strains in BHI were also plated on calibrated BHI agar. A sterile 6-mm paper disk was placed on the agar surface, and 10 μl of 200 mM potassium tellurite was added to the disk. The diameter of the growth inhibition area was measured after 24 h of incubation at 37 °C.

### Construction of *C. difficile* Strains and Plasmids

All routine plasmid constructions were carried out using standard procedures. To construct a plasmid expressing *ftsK* under the control of a P_*tet*_-inducible promoter, a fragment containing the complete gene was amplified by PCR and cloned into pDIA6103 (29). To construct the translational fusion coding for an FtsK-hemagglutinin (HA)-tagged protein, the plasmid pDIA6103-P*_tet_*-*ftsK* was amplified by inverse PCR. The PCR product was then digested by DpnI to remove the plasmid template, phosphorylated by T4 polynucleotide kinase, and ligated by T4 ligase to recircularize the plasmid. The same strategy was used for FtsK site-directed mutagenesis on T318 (threonine was replaced by an alanine to mimic nonphosphorylation). A translational *ftsK*–SNAP fusion was obtained by Gibson Assembly. SNAP coding sequence was amplified and fused to a linker in 5′ orientation (GGATCCGCAGCTGCT) using pFT58 as a template, and pDIA6103-P*_tet_*-*ftsK* was amplified by inverse PCR. A knockdown antisense system was used to deplete the *C. difficile* 630Δ*erm* strain (28) for the FtsK protein. The *ftsK* gene fragment comprising the 5′ untranslated region and the beginning of the *ftsK* coding part (−40 to +140 relative to the translational start site) was amplified by PCR and cloned into pDIA6103 in antisense orientation under the control of the ATc-inducible P*_tet_* promoter, giving pDIA7044. Plasmids generated in this study and primers are listed in supplemental Tables S1 and S2. These plasmids were transferred into *C. difficile* strains by conjugation.

### Optimized Cell Lysis and Protein Digestion

Bacterial pellets were resuspended in 100 mM ammonium bicarbonate (ABC), 50 mM DTT, 4% SDS, 1% DNase I, and 1× protease (cOmplete, Mini, EDTA-free Protease Inhibitor Cocktail; Roche) and phosphatase (Pierce Phosphatase Inhibitor Mini Tablets; Thermo Scientific) inhibitors and disrupted by ultrasonic cavitation. Protein digestion was based on the modified filter-aided sample preparation procedure (30) using 30 K Amicon Ultra-4 filtration devices (Millipore). Briefly, 4 mg protein lysate was concentrated into the filtration device at 4500*g* for 20 min and diluted with 2.0 ml of exchange buffer (EB; 100 mM ABC, 8 M urea). This step was repeated three times before adding 1 ml of EB containing 5 mM Tris(2-carboxyethyl)phosphine, 30 mM chloroacetamide, 0.3% benzonase, 0.1% DNase I, and 1 mM MgCl_2_ during 90 min at room temperature. After exchanging the buffer once with EB, the resulting concentrate was washed by three times with 50 mM ABC. After overnight incubation with sequencing grade–modified trypsin (Promega) using a ratio 1:100 for trypsin:protein, peptides were collected by centrifugation of the filter. Resulting peptides were dried by vacuum centrifugation and resuspended in 2% acetonitrile (ACN), 0.1% formic acid (FA) prior to LC–MS/MS analysis.

### Phosphopeptide Enrichment

Resulting peptides were desalted with a Sep-Pak plus C18 cartridge (Waters) and eluted with 80% ACN, 0.1% heptafluorobutyric acid (HFBA; Sigma–Aldrich), which was subsequently adjusted to 6% HFBA. Titanium dioxide (TiO_2_) beads (Sachtopore NP beads, 5 μM, 300 Å; Huntsman) were resuspended at 20 mg/ml in 30% ACN, 0.1% HFBA during 1 h, and then activated 15 min with 80% ACN/6% HFBA. Peptide solution was incubated for 30 min at room temperature with TiO_2_ using a peptide to bead ratio of 10:1. Two washes with the same buffer and one with 50% ACN and 0.1% HFBA were performed before an elution with 10% NH_4_OH. The pH was neutralized with 20% FA, and enriched peptides were freeze dried. Finally, phosphopeptides were desalted by stage-tip (31) using a C18 Empore disc and dried by vacuum centrifugation. Peptides were resuspended in 2% ACN/0.1% FA prior to LC–MS/MS analysis.

### LC–MS/MS Analysis

#### Phosphoenrichment

Tryptic peptides from phospho-enriched peptides were analyzed on a Q Exactive HF instrument (Thermo Fisher Scientific) coupled with an EASY nLC 1200 chromatography system (Thermo Fisher Scientific). The sample was loaded into a 53 cm in-house packed nano-HPLC column (inner diameter of 75 μm) with C18 resin (1.9 μm particles, 100 Å pore size, Reprosil-Pur Basic C18-HD resin; Dr Maisch GmbH) after an equilibration step in 100% solvent A (H_2_O and 0.1% FA). Peptides were eluted as previously described (27). The instrument method for the Q Exactive HF was set up in the data-dependent acquisition mode. After a survey scan in the Orbitrap (resolution of 60,000), the 10 most intense precursor ions were selected for higher energy collisional dissociation (HCD) fragmentation with a normalized collision energy set up to 27. MS/MS scans (fixed first mass of 100 *m/z*) were acquired at a resolution of 15,000. For the proteome, the automatic gain control (AGC) target and maximum injection time for the survey scans and the MS/MS scans were set to 3E6, 100 ms and 1E5, 45 ms, respectively. For the phosphopeptides, the AGC target and maximum injection time for the survey scans and MS/MS scans were set to 3E6, 50 ms and 1E6, 60 ms, respectively. The isolation window was set to 1.6 *m/z*, and normalized collision energy for HCD fragmentation was fixed to 28 for the proteome and 27 for the phosphopeptides. A minimum AGC target of 2E3 was used for an intensity threshold of 4.4E4, and a minimum of AGC target 6E3 was used for an intensity threshold of 1E5, for the proteome and phosphopeptides, respectively. Charge state screening was enabled, and precursors with unknown charge state or a charge state of 1, 7, 8 and >8 were excluded. Dynamic exclusion was enabled for 45 s and 30 s for the proteome and phosphopeptides, respectively.

#### In-Gel Digestion

Tryptic peptides from in-gel digestion were analyzed on a Q Exactive Plus instrument coupled with an EASY nLC 1200 chromatography system. Sample was loaded on an in-house packed 25 cm nano-HPLC column (inner diameter of 75 μm) with C18 resin (1.9 μm particles, 100 Å pore size, Reprosil-Pur Basic C18-HD resin) after an equilibration step in 100% solvent A (H_2_O and 0.1% FA). Peptides were first eluted using a 2 to 5% gradient of solvent B (ACN and 0.1% FA) during 5 min, then a 5 to 10% during 20 min, a 10 to 30% during 70 min, and finally a 30 to 60% during 20 min. For all, a 300 nl/min flow rate was used. The instrument method for the Q Exactive Plus was set up in the data-dependent acquisition mode. After a survey scan in the Orbitrap (resolution of 70,000), the 10 most intense precursor ions were selected for HCD fragmentation with a normalized collision energy set up to 28. Charge state screening was enabled, and precursors with unknown charge state or a charge state of 1, 7, 8 and >8 were excluded. Dynamic exclusion was enabled for 20 s.

### Data Processing for Protein Identification and Quantification

Raw files were searched using MaxQuant software (32), version 1.5.3.8 and 1.6.6.0 (for in-gel digestion) with Andromeda as a search engine (33) against an internal *C. difficile* database containing 3957 proteins (26), common known MS contaminants, and reversed sequences of all entries. Andromeda searches were performed choosing trypsin as specific enzyme with a maximum number of three missed cleavages. Possible modifications included carbamidomethylation (Cys, fixed), oxidation (Met, variable), Nter acetylation (variable), and phosphorylation (S, T, Y, variable). The mass tolerance in MS was set to 20 ppm for the first search, then 4.5 ppm for the main search, and 20 ppm for the MS/MS. Maximum peptide charge was set to seven, and seven amino acids were required as minimum peptide length. The “match between runs” feature was applied for samples having the same experimental condition with a maximal retention time window of 0.7 min. One unique peptide by protein group was required for protein quantification. A false discovery rate (FDR) cutoff of 1% was applied at the peptide, phosphopeptide, and protein levels. For in-gel analysis, we used the phospho (S/T/Y) table to extract the intensity of each phosphopeptide. A normalization step based on the intensity-based absolute quantification of the protein in each sample was performed before relative quantification. The MaxLFQ algorithm was used to provide quantified values for unmodified proteins (*i.e.*, proteins quantified from their identified and unmodified peptides) (33).

### Experimental Design and Statistical Analysis of the Proteome and the Phosphoproteome

*C. difficile* strains were cultured at 37 °C in TY for 16 h. Quantitative proteomes and phosphoproteomes were acquired from five biological replicates of five *C. difficile* strains, the 630Δ*erm* strain (WT) used as a control, and the Δ*prkC*, *CD2148*::*erm*, Δ*stp*, and double Δ*prkC CD2148*::*erm* mutant strains. Only phosphopeptides with a localization probability (LP) of their phosphorylation sites superior to 0.75 in at least one replicate were kept to compare their abundances between biological conditions (34). Intensities of proteins and phosphopeptides were normalized by condition using a median-centering function from DAPAR R package (35), and their missing values were imputed using the impute.mle function of the imp4p R package (36). This algorithm imputes values in a condition only when an intensity value has been quantified in at least one of the samples of the considered condition. The quantification profiles (modified peptide and parent unmodified protein quantified/not quantified in at least one sample of a condition) can be viewed in the “Absent/Present” columns of supplemental Table S4. For the differential analysis of one condition *versus* the WT strain, two statistical tests were used. First, a moderated *t* test was performed; thanks to the limma R package (37), to determine whether a phosphorylated peptide is significantly differentially abundant between both conditions. Moreover, phosphorylated peptides quantified in one condition and not in the other were also considered differentially abundant. Another statistical test was performed in a second step: a contrasted *t* test was performed to compare the variation in abundance of each modified peptide to the one of its parent unmodified protein using the limma R package (37). This second test allows determining whether the fold change of a phosphorylated peptide between two conditions is significantly different from the one of its parent and unmodified protein (paragraph 3.9 in Ref. (38)). An adaptive Benjamini–Hochberg procedure was applied on the resulting *p* values; thanks to the adjust.p function of R package cp4p (39), and an FDR threshold of 1% was used to select peptides that evolve differently from their parent protein. Note that this second test can be performed only when there are quantified intensity values for both the nonmodified peptide and its parent protein. Interesting cases also emerge from the absence of quantified values for the belonging protein. Consequently, differentially abundant peptides that are associated to proteins from which no fold change can be computed were also considered in the final list of differentially abundant peptides that evolve differently from their parent protein. All results can be explored in supplemental Table S4. Interestingly, most of the phosphopeptides that are concluded as differentially abundant are actually phosphopeptides quantified in a condition and not quantified in another (supplemental Tables S4 and S8). Thus, the imputation of their missing values has no influence, and no statistical test was performed for them. For Δ*prkC versus* WT, 171 are only quantified in the Δ*prkC* mutant and not in WT strain, one is quantified in both, and significantly more abundant in the Δ*prkC* mutant with a 1% FDR (supplemental Table S8). For *CD2148 versus* WT, 165 are only quantified in the *CD2148* mutant and not in WT strain, two are quantified in both, and significantly more abundant in the *CD2148* mutant with a 1% FDR (supplemental Table S8). For Δ*stp versus* WT, 358 are only quantified in the Δ*stp* mutant and not in the WT strain, 73 are quantified in both, and significantly more abundant in the Δ*stp* mutant with a 1% FDR (supplemental Table S9). For the double mutant (Δ*prkC CD2148*) *versus* WT, 102 are only quantified in the double mutant and not in the WT strain, five are quantified in both, and significantly more abundant in the double mutant with a 1% FDR (supplemental Table S4).

### Motif Analysis of Phosphopeptides

Visualization of motifs was performed using the ggseqlogo R package (40). Motif enrichment analysis was performed using the rmotifx R package (41) using a background of peptides with random amino acid sequences centered on S, T, or Y amino acids respecting the measured proportions in the WT strain.

### Protein–Protein Interaction Networks

Protein–protein interaction networks have been performed using Cytoscape software and the stringApp (42) and Omics Visualizer apps (43). Widths of the edges are functions of the combined score of STRING v11 database (44): it reflects the overall confidence that we can have in each interaction. Functional categories have been defined from the analysis of protein functions.

### Analysis of Protein Functions

Proteins were classified into functional categories according to their annotated functions in the GeneBank database, publications, and by homology/functions according to the Gene Ontology (45), the Conserved Domain (https://www.ncbi.nlm.nih.gov/Structure/cdd/cdd.shtml), and the Kyoto Encyclopedia of Genes and Genomes Pathway (http://www.genome.jp/kegg-bin/show_organism?org_sco) databases. Enrichments of Gene Ontology terms, DAVID keywords, and Kyoto Encyclopedia of Genes and Genomes pathways were performed using hypergeometric tests; thanks to DAVID software (46). The set of all identified proteins related to phosphosites was used as background for the statistical tests. A *p* value threshold of 1% was used to select enriched terms/keywords/pathways.

### Phos-Tags and Western Blots

For Phos-Tag fluorescence, SDS-PAGE gels were incubated in a solution containing Phos-Tag (Phos-Tag Phosphoprotein Gel Stain; ABP Biosciences) and then washed in Phos-Tag Phosphoprotein Destain Solution (ABP Biosciences). Phosphorylated proteins specifically stained were detected using a fluorescence imaging scanner. Phos-Tag acrylamide and Western immunoblotting were carried out using standard methods. Phos-Tag acrylamide is an electrophoresis technique capable of separating phosphorylated and nonphosphorylated forms based on phosphorylation levels (47). Proteins were run on an 8% SDS-PAGE supplemented with 50 μM of Phos-Tag acrylamide (catalog no.: AAL-107; Wako) and 100 μM MnCl_2_. Proteins were electrophoresed and transferred to polyvinylidene difluoride membranes. After blocking with nonfat milk in Tris-buffered saline with Tween-20 buffer, primary antibodies against the HA epitope (Osenses) were added. The washed membranes were incubated with appropriate secondary antibodies coupled to horseradish peroxidase that were detected by an enhanced chemiluminescence system. To detect phosphorylated threonine, total cellular extracts were run on an SDS-PAGE. Proteins were then transferred to polyvinylidene difluoride membranes, and phosphothreonine (pT) was detected using an anti-pT antibody (Cell Signaling Technology).

### *In Vitro* Phosphorylation Assay

Purified KDs of PrkC (PrkC-KD, amino acids 1–335) (pDIA6406) and CD2148 (CD2148-KD, amino acids 1–278) (pDIA6407) (27) were incubated in a kinase buffer containing 50 mM Tris (pH 7.5) and 5 mM MgCl_2_. We also cloned a complete copy of *CD2148* using oligonucleotides SAT119 and SAT120 between the BamHI and KpnI sites of pQE30 to give plasmid pDIA7208. This plasmid carried CD2148 encoding a protein fused to a His_6_ tag expressed under the control of an IPTG-inducible promoter. His_6_-tagged proteins were produced in *E. coli* strain M15pRep4, and the protein was purified as previously published (27). His_6_-CD2148 (0.1 μM) was incubated in the presence of 5 mM ATP and γ^32^P-ATP in the kinase buffer. His_6_-CD2148-KD (10 μM) and His_6_-PrkC-KD (10 μM or 1 μM) were incubated alone or in combination in the kinase buffer. His_6_-Spo0A (10 μM) was incubated alone or in combination with His_6_-PrkC-KD or His_6_-CD2148-KD (1 μM or 2 μM) in the kinase buffer. In both cases, the reactions were initiated by the addition of 5 mM ATP and incubated at 37 °C for 90 min. Reactions were stopped with the addition of SDS–Laemmli sample buffer, and proteins were subjected to Phos-Tag, SDS-PAGE, or Western immunoblotting against pT antibodies or phosphoserine (pS) antibodies (Sigma). His_6_-PrkC-KD and/or His_6_-CD2148-KD was used to phosphorylate His_6_-Spo0A or His_6_-CD2148-KD *in vitro* as described previously. The protein mixture was separated by SDS-PAGE and stained with Coomassie blue. The band corresponding to His_6_-PrkC-KD, His_6_-CD2148-KD, or His_6_-Spo0A was excised and subjected to tryptic digestion as previously described (48, 49). Resulting peptides were dried by vacuum centrifugation and resuspended in 2% ACN and 0.1% FA prior to LC–MS/MS analysis.

### Phase Contrast and SNAP Labeling Microscopy

For phase contrast microscopy, *C. difficile* strains were cultured for 5 h in TY (with antibiotics and inducers when needed) at 37 °C. Cells were visualized using Zeiss Axioskop microscope and analyzed using the ImageJ software. For SNAP labeling, strains were grown 3 h in TY. The expression of the SNAP^Cd^–PrkC, SNAP^Cd^–PrkCΔSGN, SNAP^Cd^–PrkCΔext, and FtsK–SNAP^Cd^ fusions was induced with 50 ng/ml of ATc for 2 h. The TMR-Star substrate (New England Biolabs) was added at 250 nM, and the mixture was incubated for 30 min in the dark under anaerobiosis. Cells were then collected by centrifugation, washed, and resuspended in PBS. Cell suspension (3 μl) was mounted on 1.7% agarose-coated glass slides. The images were taken with exposure times of 600 ms for autofluorescence and 800 ms for SNAP using a Nikon Eclipse TI-E microscope (100× objective) and captured with a CoolSNAP HQ2 Camera. The images were analyzed using ImageJ.

## Results

### Identification of Phosphopeptides for *C. difficile*

The substoichiometric nature of protein phosphorylation requires an efficient method for enrichment of phosphopeptides prior to MS analysis. We aimed to establish an efficient, robust, and reproducible enrichment of phosphopeptides from *C. difficile*. We harvested cells at 4, 16, and 24 h and tested various conditions of cell lysate preparation: chemical lysis using chaotropic agents (urea) or detergents (SDS, Triton, and deoxycholic acid) in the presence or the absence of benzonase and DNAse. Different amounts of starting material ranging from a few 100 μg to 8 mg of proteins were then digested with trypsin. Phosphopeptides were enriched *via* a TiO_2_ phosphopeptide enrichment procedure using two different peptides to bead ratios (10:1 and 4:1) and different incubation times. The performance of trifluoroacetic acid and HFBA for this step was also tested. After LC–MS analysis of the phosphopeptides detected, the highest number of phosphopeptides was defined in the following optimized experimental conditions (supplemental Table S3 and supplemental Fig. S1). After 16 h of growth in TY medium, an extraction was performed in an SDS buffer in the presence of DNAse and benzonase. A protein to bead ratio of 10:1 for TiO_2_ enrichment in HFBA was used with an incubation of 30 min. The enriched phosphopeptides were then analyzed by nano LC–MS/MS.

**Fig. 1:**
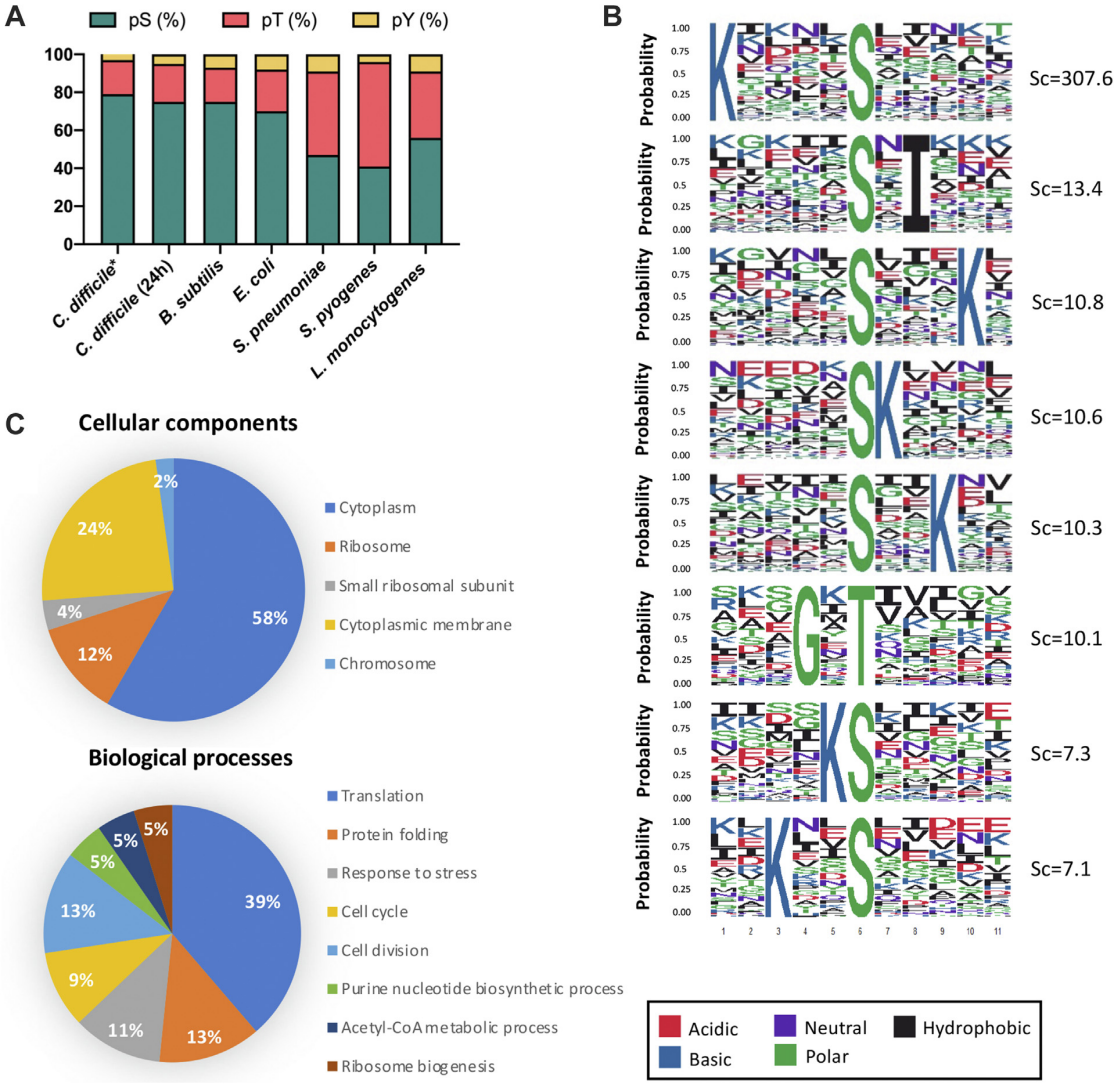
**Overview of the *Clostridioides difficile* 630Δ*erm* phosphoproteome.***A*, distribution of phosphorylated residues across all phosphoproteins found in *C. difficile* compared with other Gram-positives (*Bacillus subtilis*, *Streptococcus pneumoniae*, *Streptococuus pyogenes*, and *Listeria monocytogenes*) and Gram-negative (*Escherichia coli*) bacteria (14, 20, 50, 77). pS means phosphoserine, pT phosphothreonine, and pY phosphotyrosine. *B*, significantly enriched motifs in the phosphopeptides detected in the *C. difficile* WT strain 630Δ*erm* (*p* < 1e-6). A background of 100,000 random sequences around the S, T, or Y amino acids was used to estimate the motif scores (Sc). The higher the motif scores, the more the motifs are overrepresented against the background. *C*, Gene Ontology distribution of identified phosphoproteins with respect to biological processes and cellular components with the DAVID functional annotation tools. The percentages indicate the proteins that belong to each category. S, serine; T, threonine; Y, tyrosine.

Our optimized strategy provided one of the largest phosphoproteome of the *C. difficile* strain 630Δ*erm* with the identification of 1340 phosphopeptides (pS, pT, and phosphotyrosine [pY]) (supplemental Fig. S1 and supplemental Table S3). When filtering on the Andromeda LP (34), 1061 phosphopeptides (LP > 0.75) were identified corresponding to 504 proteins (supplemental Fig. S1 and supplemental Table S3). Similar types of results have been recently obtained at 24 h in a phosphoproteomic study with the same *C. difficile* strain (22). The average abundances of pS and pT is 77% and 18%, respectively, and as expected, pY are less abundant (5%) (Fig. 1*A* and supplemental Table S3) in agreement with the distribution observed in *C. difficile* at stationary phase (22) and similar to the distributions found in *B. subtilis* and *E. coli* (14, 20, 50) (Fig. 1*A*). A few enriched motifs around phosphorylated S residues can be detected with the presence of lysine residues at position −5, −3, −1, +1, +3, or +4 and of an isoleucine at +2 with respect of pS, whereas a unique enriched motif (GXT) is found for pT (Fig. 1*B*). The biological functions of the phosphoproteins identified in *C. difficile* were classified based on their Gene Ontology terms. Most identified phosphoproteins were assigned to the cytoplasm, but 24% was also associated with the cytoplasmic membrane (Fig. 1*C*, *top*). As observed in another phosphoproteome of *C. difficile* (22), many phosphorylated peptides belong to proteins involved in translation, stress response including chaperones, cell division, envelope homeostasis, and carbon metabolism including enzymes of glycolysis and transporters belonging to the phosphotransferase system (PTS) (Fig. 1*C*, *bottom*). Known kinase substrates were also identified: (i) the anti–anti sigma factor RsbV on S57 as previously observed (22, 51), likely by the anti–sigma and kinase factor, RsbW, which was also phosphorylated on S89; (ii) the HPr protein of the PTS on S45 in agreement with data obtained *in vivo* (22), and *in vitro* using purified HPr and HPr-kinase proteins (52) and on S11 as found in *B. subtilis* (22, 53, 54). As previously observed in other firmicutes (20), we found that the phosphoglutamine mutase, GlmM, which is involved in PG biosynthesis, was phosphorylated on S100 located at its active site and that the transition phase regulator, CodY, was phosphorylated on several residues including S216 shown as phosphorylated in *Bacilli* (55). Interestingly, toxin A, TcdA, is phosphorylated on multiple S residues (supplemental Table S3). The identification of more than 1000 phosphosites localized in S and T residues suggests that STK signaling pathways play an important role in the physiology of this human enteropathogen.

### Autophosphorylation of the Hanks Kinases of *C. difficile*

**Fig. 2:**
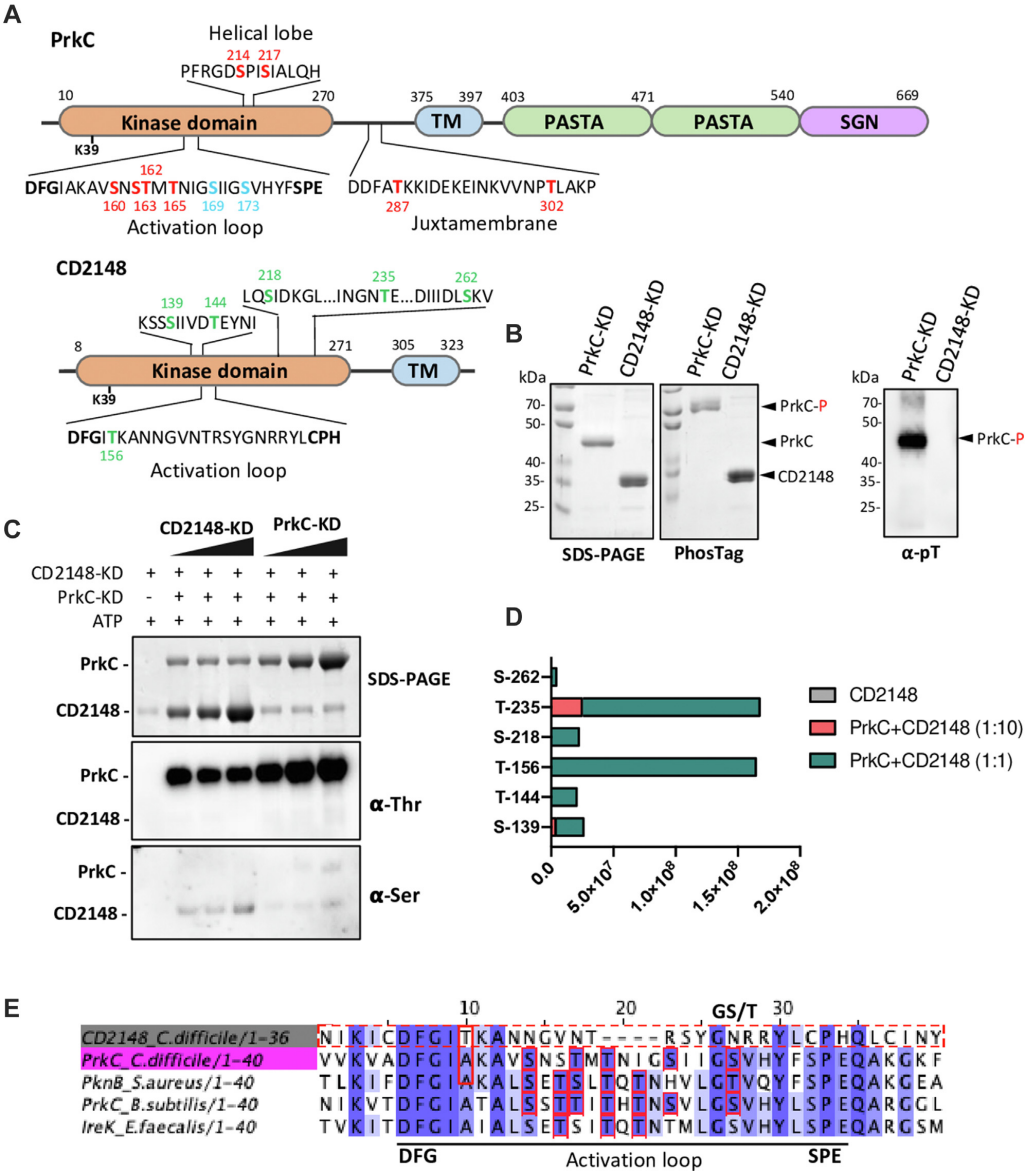
**Phosphorylation of STKs *in vivo* and *in vitro*.***A*, a schematic presentation of PrkC and CD2148 containing location of the different domains is indicated. The kinase domain (KD) containing the activation loop, the transmembrane (TM) region, the PASTA domains, and the Ser-Gly-Asn (SGN) rich segment are indicated. Phosphoresidues detected *in vivo* and *in vitro* are indicated in *red*, and phosphoresidues detected only *in vivo* are indicated in *blue* (supplemental Table S3) and only *in vitro* in *green*. *B*, *in vitro* phosphorylation of purified PrkC-KD and CD2148-KD proteins in presence of 5 mM ATP analyzed by SDS-PAGE, Phos-Tag, and Western blot using anti-pT antibodies (⍺-pT). *C*, *in vitro* phosphorylation of CD2148-KD in the presence of PrkC-KD analyzed by Western blot using anti-pT (⍺-Thr) and anti-pS (⍺-Ser) antibodies. *D*, mass spectrometry detection of CD2148 phosphorylation sites after *in vitro* phosphorylation reactions performed without or with PrkC-KD at ratio 1:1 and 1:10. *E*, multiple sequence alignment of activation loop of PrkC (*pink*) and CD2148 (*gray*) with homologous STKs: *Staphylococcus aureus* PknB, *Bacillus subtilis* PrkC, and *Enterococcus faecalis* IreK. Conserved residues are marked in *blue color*, and phosphorylated residues are shown in *red boxes*. The T/S amino acid of the GT/S motif is indicated by a *black arrow*. KD, kinase domain; PASTA, penicillin-binding and STK-associated; pS, phosphoserine; pT, phosphothreonine; S, serine; STK, serine/threonine kinase; T, threonine.

Since STKs are classically able to autophosphorylate (4), we also analyzed their phosphorylation. In the WT strain, PrkC was phosphorylated on 12 amino acids (supplemental Tables S3 and S4), nine being common with the recent *C. difficile* phosphoproteome (22). By contrast, although the protein was detected, no phosphopeptides were identified for CD2148, which is in agreement with the other phosphoproteomic study (22). The common regulatory mechanism for activation of STKs is the autophosphorylation on S and T residues of a canonical kinase segment known as the activation loop, starting right after the conserved DFG motif and ending with the SPE motif (56, 57, 58). PrkC can be phosphorylated *in vivo* on six different p-sites on the activation loop but not at the same time (S160, T162, T163, T165, S169, and S173), and two Ts in the juxtamembrane domain (T287 and T302) (Fig. 2*A* and (22)). By contrast, two S in the helical lobe (S214 and S217) were detected as phosphorylated only in our phosphoproteome. Analysis of the full KD of CD2148 revealed the presence of Hanks signature motifs suggesting that CD2148 is a Hanks-type STK (supplemental Fig. S2, *roman numbers* I–XI) (59). CD2148 shares similarities with the catalytic domain of PASTA-STKs (26% with *C. difficile* PrkC, 32% with *B. subtilis* PrkC, and 26% with *S. pneumoniae* StkP) and with the cytoplasmic YbdM (22%) or the transmembrane YabT (25%) kinases of *B. subtilis*.

We then wondered if PrkC and CD2148 might autophosphorylate *in vitro*. Purified KDs of PrkC (PrkC-KD) and CD2148 (CD2148-KD) were incubated in the presence of ATP and Mg^2+^, and the phosphorylation was detected using either a Phos-Tag gel or an anti-pT antibody. As observed *in vivo*, autophosphorylation was only detected for PrkC under the conditions tested (Fig. 2*B*). LC–MS/MS analysis yielded phosphopeptides with 10 phosphorylation sites in PrkC, eight of them were identified *in vivo* (Fig. 2*A*, *red numbers* and supplemental Fig. S3*B*). Since the CD2148-KD protein did not autophosphorylate *in vitro* (Fig. 2*B*), we tested a full-length purified CD2148 protein to exclude that the deletion of the transmembrane segment anchoring the protein in the cytoplasmic membrane (27) has an impact on its phosphorylation. We failed to detect phosphorylation of CD2148 in the presence of γP^32^ATP, whereas PrkC-KD was phosphorylated (supplemental Fig. S3*A*). We then decided to test if the phosphorylation of each kinase can be increased or modified in the presence of the other kinase. The addition of CD2148 to PrkC did not modify the sites of phosphorylation as determined by LC–MS/MS analysis (supplemental Fig. S3*C*). While we failed to detect a phosphorylation in the presence of CD2148-KD alone using both anti-pT and anti-pS antibodies (Fig. 2*C*), we observed a signal of phosphorylation of CD2148-KD in the presence of PrkC using an anti-pS antibody. LC–MS/MS analysis yielded phosphopeptides with six phosphorylation sites in CD2148-KD in the presence of PrkC, three S, and three T residues (Fig. 2*D*). Interestingly, T156 located in the activation loop was phosphorylated in the presence of PrkC (Fig. 2*A*). Sequence alignment of activation loop of PrkC and CD2148 with homologous STKs revealed that PrkC is phosphorylated on several residues, including the T located at the conserved GT/S motif (57, 60). In contrast, CD2148 lacks both the GT/S motif and the conserved SPE motif at the end of the activation loop (Fig. 2*E*) and is phosphorylated on a nonconserved T residue of this loop (Fig. 2*E*). Despite the fact that a phosphorylation of CD2148 was not detected *in vivo* (supplemental Table S3, (22)), we observed multiple phosphorylations of CD2148-KD *in vitro* in the presence of PrkC. The role of PrkC might be either to stabilize CD2148∼P, to stimulate CD2148 autophosphorylation, or to directly phosphorylate CD2148. Crossphosphorylation between different kinases of *B. subtilis* has been observed *in vitro* (61), PrkC exhibiting the highest capacity to phosphorylate other kinases. This result also suggests that the phosphorylation of candidate targets of CD2148 could be modulated by PrkC.

### PASTA Domains are Not Necessary for PrkC-Dependent Phosphorylation and Septal Localization *in Vivo*

**Fig. 3:**
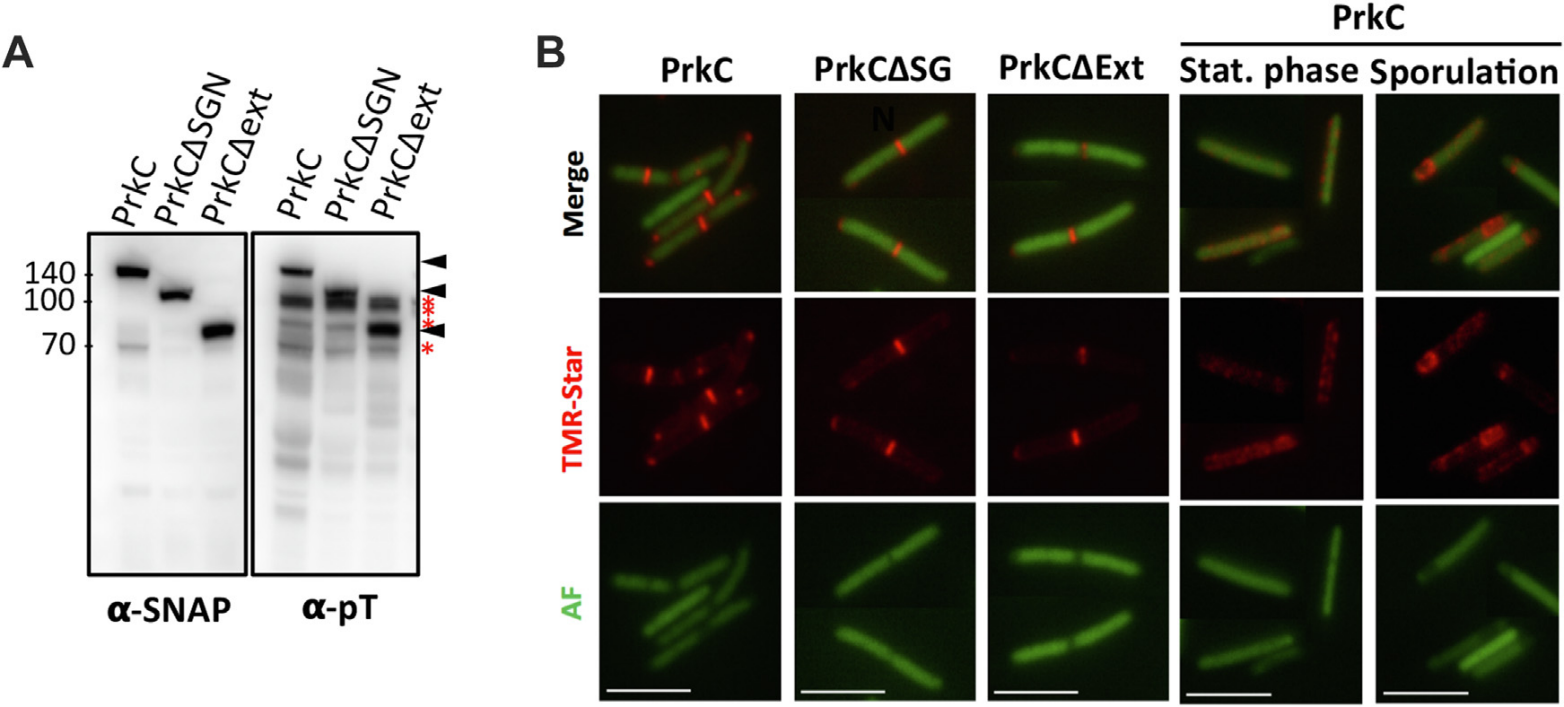
**PASTA domains are not necessary for PrkC autophosphorylation and for its septal localization.***A*, Western blots of whole-cell lysates from cells expressing SNAP–PrkC or truncated versions were probed with anti-SNAP antibodies (⍺-SNAP) and anti-pT antibodies (⍺-pT). The PrkC phosphorylation signal is indicated by a *black arrow*, and unknown phosphorylated proteins are indicated in *red asterisk* (∗). *B*, localization of full-length and truncated SNAP–PrkC. *Merge images*, TMR-Star fluorescent signal and autofluorescence. The scale bar represents 5 μm. PASTA, penicillin-binding and STK-associated; pT, phosphothreonine.

There are increasing evidence that PASTA-STKs can respond to cell wall–derived signals *via* their PASTA domains. In *Enterococcus faecalis*, PASTA domains are required for the autophosphorylation of the kinase IreK in response to cell wall stress (62). In *S. pneumoniae* StkP and *Mycobacterium tuberculosis* PknB, PASTA domains have been shown crucial for their proper localization at midcell (63, 64, 65). To test if the PASTA repeats are required for PrkC autophosphorylation or localization, we fused SNAP with truncated PrkC proteins lacking either the SGN-rich region (26) or the full extracellular domain. Cells expressing these SNAP–PrkC proteins under the control of a P_*tet*_-inducible promoter (50 ng/ml of ATc) were analyzed by Western blot using antibodies against SNAP to determine their level of synthesis and with antibodies against pT to detect the phosphorylation of PrkC *in vivo*. All truncated SNAP–PrkC fusions were produced at similar levels, and these proteins were able to autophosphorylate *in vivo* and to transfer their phosphate to target proteins (Fig. 3*A*). Using fluorescence microscopy, we also showed that both truncated SNAP–PrkC proteins were located at the septum of the cell during exponential growth as observed for the full-length protein (Fig. 3*B*) (27). Thus, the PASTA domains and the SGN motif are not necessary for PrkC-dependent phosphorylation or for its septal localization as observed for the PASTA kinase of *B. subtilis* (66). In addition, we observed that the SNAP–PrkC fusion delocalized during stationary phase (Fig. 3*B*). PrkC was also detected associated with the forespore during sporulation (Fig. 3*B*). This suggests that PrkC kinase has several cellular localizations and can play a role in different growth phases and during sporulation similar to PrkC of *B. subtilis* (66).

### Differential Phosphorylation in Mutants Inactivated for Genes Encoding STKs or STP and Identification of Candidate Substrates

**Fig. 4:**
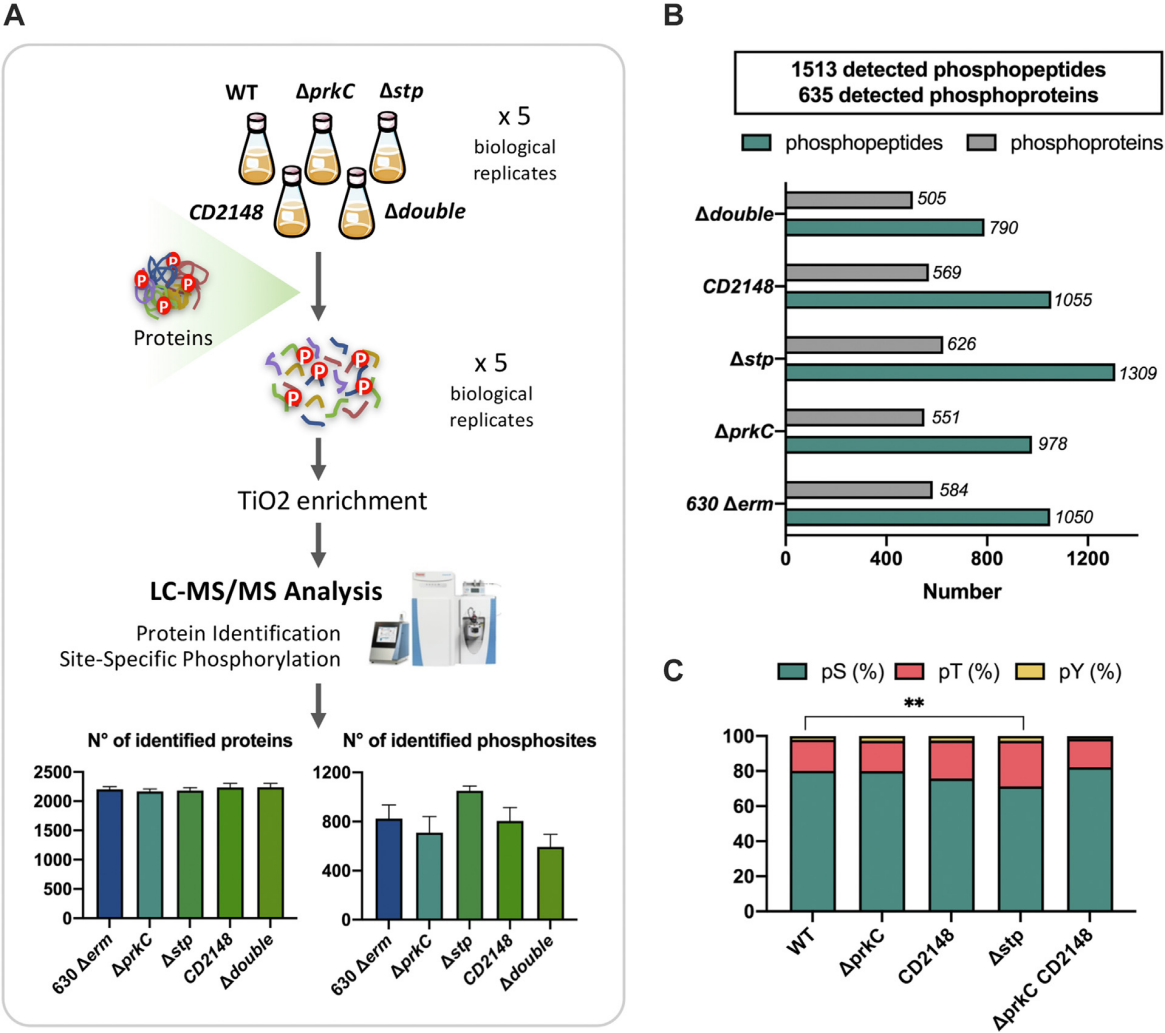
**Phosphoproteomic analysis of STKs and STP mutants.***A*, schematic workflow for quantitative phosphoproteomic analysis of the WT (630Δ*erm*) strain and the Δ*prkC*, *CD2148*, Δdouble (Δ*prkC CD2148*), and Δ*stp* mutants. Five replicates were performed for each strain. Extracted protein samples were digested with trypsin followed by a phosphopeptide TiO_2_ enrichment using our optimized protocol. The resulting samples were analyzed by nano LC–MS/MS on a Q Exactive HF mass spectrometer, and average number of identified proteins and phosphosites is shown at the *bottom*. *B*, number of identified proteins (2493 in total) and phosphoproteins for each strain (for a total number of 1735 identified phosphopeptides, 1513 have an LP > 0.75 corresponding to 635 phosphoproteins). *C*, distribution of phosphorylation sites across all phosphoproteins found in each strain. pS means phosphoserine, pT phosphothreonine, and pY phosphotyrosine. A significantly different proportion of pT is observed between the Δ*stp* mutant and the WT strain (*p* < 5%). LP, localization probability; STK, serine/threonine kinase; STP, serine/threonine phosphatase; TiO_2_, titanium dioxide.

**Fig. 5:**
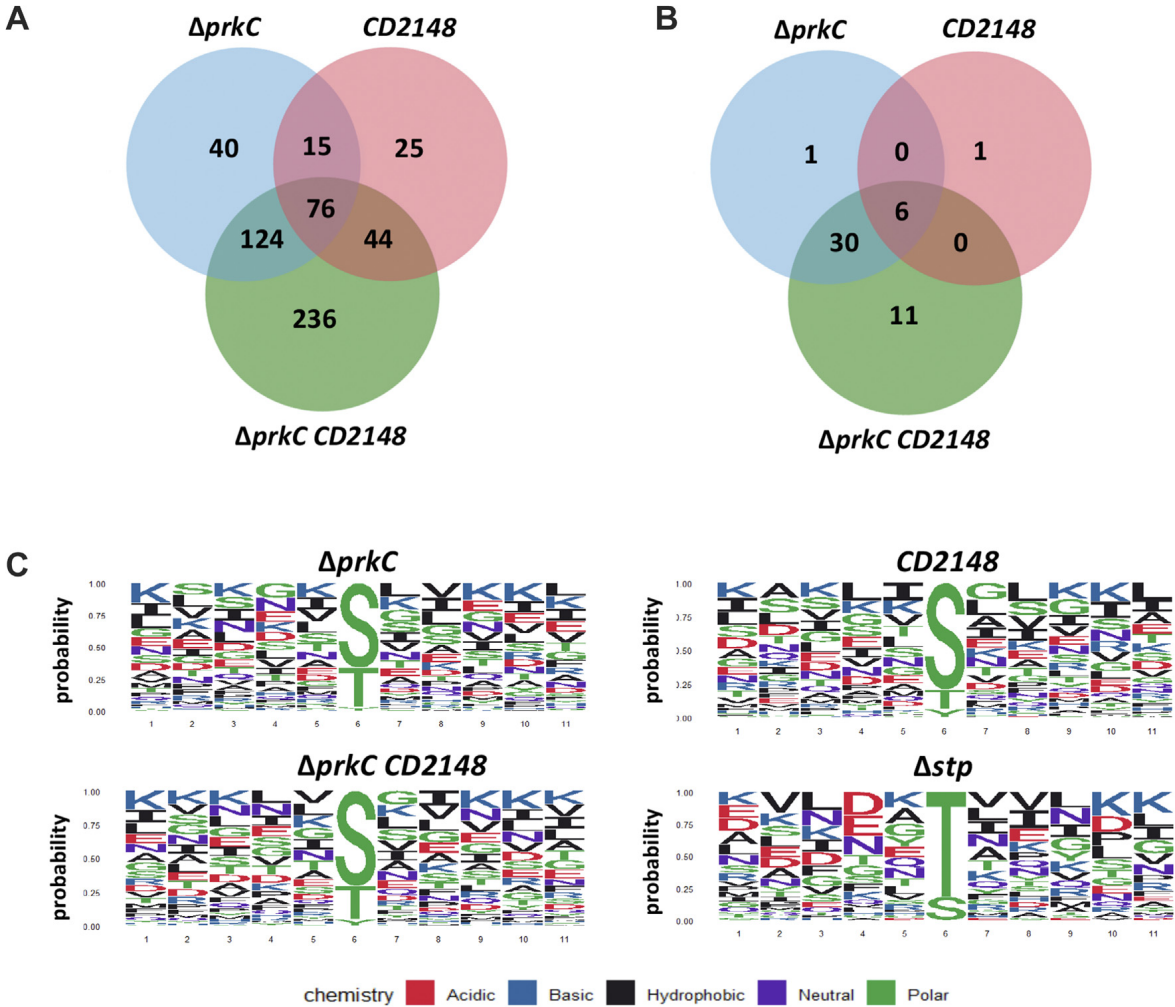
**Identified phosphorylation changes in *Clostridioides difficile* mutants inactivated for the genes encoding STK or STP.** Venn diagram showing overlaps of the phosphopeptides (LP > 0.75) significantly less abundant in the kinase mutants (Δ*prkC*, *CD2148*, or double) compared with WT strain (*A*) or also more abundant in the Δ*stp* mutant compared with WT strain (*B*) (supplemental Table S7). *C*, phosphorylation site motif analysis highlighting the distribution of amino acids at positions adjacent to the pT or pS in phosphopeptide lists. Phosphopeptides less abundant in the Δ*prkC* (*top left*), *CD2148* (*top right*), or the double mutants (*bottom left*) than in the WT strain. In *bottom right*, motif analysis of phosphopeptides more abundant in the Δ*stp* mutant among the phosphopeptides less abundant in the Δ*prkC*, *CD2148*, or double mutants than in the WT strain. The ggseqlogo R package was used. LP, localization probability; pT, phosphothreonine; pS, phosphoserine; STK, serine/threonine kinase; STP, serine/threonine phosphatase.

Though two phosphoproteomes for *C. difficile* have been reported (supplemental Table S3 and (22)), it is unknown how kinases contribute to the observed phosphorylation patterns. Identification of direct substrates of PrkC and/or CD2148 can provide insight into the mechanisms by which protein phosphorylation by each kinase controls cell physiology. To try to identify kinase or phosphatase substrates, we performed proteome and phosphoproteome of the five *C. difficile* strain 630Δ*erm* as a control and the Δ*prkC*, *CD2148*, Δ*stp*, and double Δ*prkC CD2148* mutants after 16 h of growth in TY (supplemental Fig. S1, *B* and *C*). The different strains grew similarly in TY medium (supplemental Fig. S1, *B* and *C*). The data were acquired from five biological replicates of five *C. difficle* strains. Phosphopeptides were enriched using our optimized protocol and analyzed *via* LC–MS/MS leading to a high reproducibility of results (Fig. 4*A*). Combining the data obtained for the WT strain and all the mutants, we identified 2493 proteins and 1735 phosphopeptides including 1513 with an LP >0.75 (Fig. 4*B*, supplemental Tables S4 and S5). The quantity of a few proteins significantly changes between one of the mutant strains and the WT strain (Fig. 4*A* and supplemental Table S6) suggesting that PrkC and CD2148 play only a minor role in the control of protein production or degradation. However, the number of identified phosphopeptides differs between strains (Fig. 4*B* and supplemental Table S4). The potential targets of PrkC/CD2148 and STP should then show opposite behavior. In the Δ*prkC* and double Δ*prkC CD2148* mutants, less phosphopeptides were detected compared with the WT strain, whereas less differences were observed for the *CD2148* mutant (Fig. 4*B*). In contrast, an increase in the number of identified phosphopeptides was observed in the Δ*stp* mutant (Fig. 4*B*). To help to define candidate substrates for each kinase, we generated a Venn diagram to examine the overlap of phosphopeptides and phosphoproteins with decreased phosphorylation among the single and double mutants inactivated for STKs (Fig. 5, *A* and *B* and supplemental Fig. S4, *E* and *F*). The phosphorylation of 236 phosphopeptides (supplemental Table S7) was significantly less abundant only in the double *CD2148* Δ*prkC* mutant suggesting that several phosphorylation events might strictly require both Hanks kinases. About 124 and 44 phosphopeptides were specific for PrkC and CD2148, respectively, and 76 phosphopeptides were significantly less abundant in the three mutants (Figs. 5*A* and supplemental Fig. S4*E*). The two kinases could function together for some targets in agreement with the involvement of PrkC to detect an efficient phosphorylation of CD2148 *in vitro*. Interestingly, we also observed that a few candidate targets specific of PrkC were more phosphorylated in the CD2148 mutant as observed for CwlA (supplemental Table S8) (27). Among the peptides differentially phosphorylated in the STK mutants compared with the WT strain, we searched for conserved sequence motifs using ggseqlogo R package (40). Although no specific signatures were detected, an enrichment for S was observed among the sites phosphorylated under the control of CD2148 (Fig. 5*C*). Indeed, the phosphorylation sites specifically dependent on PrkC correspond to pS for 59% of them, whereas those detected as specifically dependent on CD2148 are mainly pS (82%) with only two pT detected (supplemental Table S7). All these results suggest that PrkC and CD2148 are involved directly or indirectly in the phosphorylation of common and specific proteins.

**Fig. 6:**
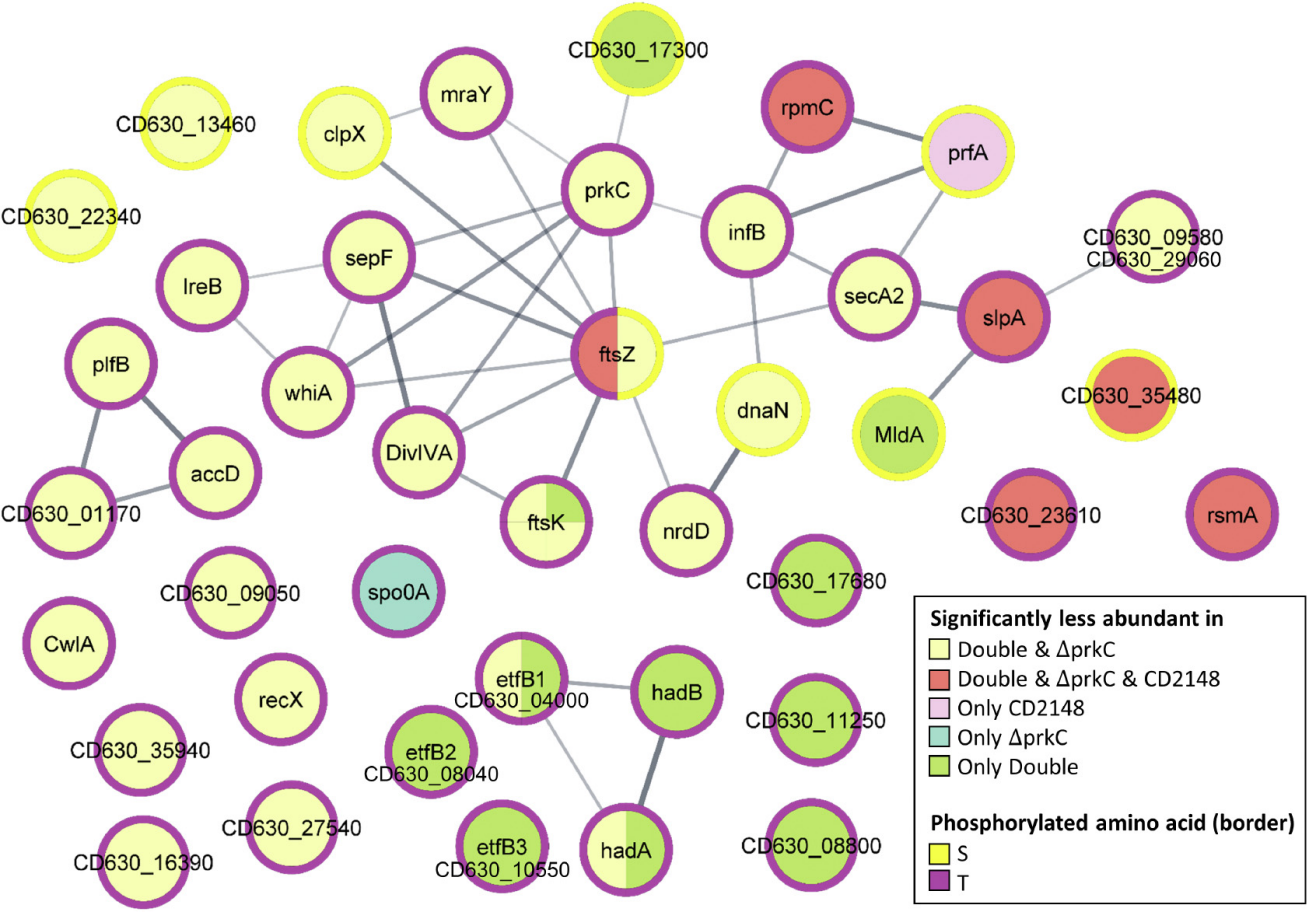
**Protein targets of STK and STP in *Clostridioides difficile*.** Protein–protein interaction network of proteins with phosphopeptides less abundant in the STK mutants compared with the WT strain and more abundant in the Δ*stp* mutant compared with the WT strain. STK, serine/threonine kinase; STP, serine/threonine phosphatase.

Among the 1309 phosphopeptides detected in the Δ*stp* mutant, a significant enrichment for pT is observed compared with the WT strain (Fig. 4*C*). About 431 phosphopeptides were more abundant in the Δ*stp* mutant than in the WT strain, whereas 358 phosphopeptides were found only in the Δ*stp* mutant (supplemental Table S9) as recently observed in *Staphylococcus aureus* (19). Since STP activity may mask the effect of STKs by dephosphorylation, we can detect in the Δ*stp* mutant phosphorylation events that are normally low in abundance or unstable. In addition, 67% of the phosphosites more abundant in the Δ*stp* mutant were less abundant in at least one mutant inactivated for a gene encoding STK (Fig. 5*B* and Table 1). Indeed, 30 phosphopeptides less abundant both in the Δ*prkC* and double mutants were more phosphorylated in the absence of STP (Fig. 5*B* and Table 1). Interestingly, among the phosphopeptides less abundant in both the *CD2148* and double mutants, increased phosphorylation was not detected in the Δ*stp* mutant (Fig. 5*B*). It is worth noting that more than 80% of the phosphosites phosphorylated under the control of STKs and dephosphorylated under the control of STP were pT (Figs. 5*C* and 6 and Table 1). A similar over-representation of pT among the Stk1/Stp targets has also been observed in *S. aureus* (19). The very few pT identified among the CD2148-dependent phosphosites could explain the lack of STP modulation of the CD2148 targets. STP might then be a phosphatase associated rather with PrkC than with CD2148. Another level of complexity is the presence of two Hanks-type kinases with possible relationships between them as suggested by our *in vitro* data (Fig. 2*C*). Later, we will focus our analysis on (i) pathways and cellular processes over-represented in the comparative phosphoproteome or known as controlled by STK-dependent phosphorylations in other firmicutes (6, 7, 8), (ii) functions related to the phenotypes of the Δ*prkC* or *CD2148* mutant (26, 27), and/or (iii) proteins whose phosphorylation decreased in the Δ*prkC* and double mutants or in the *CD2148* and double mutants with a highlight for the phosphopeptides more abundant in the Δ*stp* mutant.

**Table 1:** Phosphosites of proteins phosphorylated in the presence of PrkC and/or CD2148 that are more phosphorylated in the Δ*stp* mutant

ID gene	Gene names	Description	Phosphopeptides	S/T	P-site
Less abundant in the Δ*prkC* and double mutants but not in the *CD2148* mutant					
CD0002	*dnaN*	DNA polymerase III β subunit	S(0.001)C**S**(0.999)VDSLTENIK	S	182
CD1346		Uncharacterized protein	KGY**S**(1)ILKDEAK	S	99
CD2234		Transcriptional regulator, Crp family	VIIFNNL**S**(0.831)T(0.169)K	S	19
CD2646	*ftsZ*	Cell division protein FtsZ	QAIQSPLLET(0.051)**S**(0.949)IQGAK	S	254
CD3304	*clpX*	ATP-dependent Clp protease, ATP-binding subunit ClpX	KS(0.04)S(0.167)**S**(0.793)KDIEIQK	S	102
CD0108	*nrdD*	Anaerobic ribonucleoside triphosphate reductase	VLSNVCGS(0.061)E**T**(0.902)VS(0.037)GR	T	471
CD0117		Putative ferredoxin/flavodoxin oxidoreductase, β-subunit	NAE**T**(1)QGYPIR	T	153
CD0395	*hadA*	Isocaprenoyl-CoA:2-hydroxyisocaproate CoA-transferase	DKPGFDY**T**(1)AYFAR	T	143
CD0400	*etfB1*	β-subunit of electron transfer flavoprotein	LDIPQV**T**(1)YVQDFK	T	143
CD0759	*pflB*	Pyruvate formate lyase	EQQLDVINR**T**(1)FHGK	T	737
CD0905		Putative phage protein	NFDK**T**(1)VEYTK	T	528
CD2906/CD0958		Putative phage protein	AITGA**T**(1)IAGGIGLVAGNIGAK	T	156
CD1135	*cwlA*	SH3-domain endopeptidase	FIHCSGTQ**T**(1)NPNKVK	T	405
CD1283	*ireB*	Small conserved protein (UPF0297)	DLDY**T**(1)MKFEGIPEDR	T	8
CD1309	*infB*	Translation initiation factor IF-2	DEMLKS(0.026)**T**(0.974)QR	T	426
CD1324	*ftsK*	DNA FtsK/SpoIIIE translocase	SAIDMMTDEVDD**T**(0.943)T(0.057)INK	T	217
CD1324	*ftsK*	DNA FtsK/SpoIIIE translocase	KSAIDMMTDEVDDT(0.01)**T**(0.99)INK	T	218
CD1324	*ftsK*	DNA FtsK/SpoIIIE translocase	KVDIAKPNLNIEK**T**(1)QPMSIVAEPVNEDYSNYK	T	318
CD1639		Tellurite-associated resistance protein	ALDDK**T**(1)NELLLR	T	303
CD1937	*accD*	Acetyl-coenzyme A carboxylase carboxyl transferase β-subunit	VIEQ**T**(1)INQK	T	241
CD2525	*recX*	Regulatory protein RecX	IVN**T**(1)NVNLNK	T	118
CD2578	*prkC*	Serine/threonine protein kinase	NIDLDFIKEYDDFA**T**(1)KK	T	287
CD2578	*prkC*	Serine/threonine protein kinase	VVNP**T**(1)LAKPAPEK	T	302
CD2619	*divIVA*	Cell-division initiation protein DivIVA	CVNAFEG**T**(0.959)T(0.041)VYNYSNDEAATTLE	T	160
CD2622	*sepF*	Cell division protein SepF	IVNLHT(0.054)**T**(0.94)S(0.006)QMK	T	56
CD2654	*mraY*	UDP-MurNAc-pentapeptide phosphotransferase	DDGPQ**T**(1)HLAK	T	46
CD2754		Diguanylate kinase signaling protein	GLTDGL**T**(1)GVVTR	T	360
CD2792	*secA2*	Protein translocase subunit SecA 2	TSSNEN**T**(1)AIESK	T	573
CD3397	*whiA*	Probable cell division protein WhiA	IVNCE**T**(1)ANLSK	T	236
CD3594		Uncharacterized protein	NEELFR**T**(1)R	T	172
Less abundant in all mutants (Δ*prkC*, *CD2148*, and double mutants)					
CD3548		YaaT-like protein involved in sporulation or related to DNA replication	DQ**S**(1)LSLNPTK	S	186
CD0080.1	*rpmC*	50S ribosomal protein L29	FQLATGQLEN**T**(1)AR	T	40
CD2361		ABC-type transport system, nitrate/sulfonate/taurine ATP-binding protein	DINK**T**(1)FVNNR	T	11
CD2646	*ftsZ*	Cell division protein FtsZ	SSLN**T**(0.834)T(0.166)VK	T	340
CD2793	*slpA*	Precursor of the S-layer proteins	**T**(1)APLLLTSK	T	444
CD3523	*ksgA*	16S rRNA dimethyladenosine transferase	LSSHNA**T**(1)KEVVQK	T	10
Less abundant only in the double mutant but not in the Δ*prkC* or *CD2148* mutant					
CD1730		Iron–sulfur cluster carrier protein	AYS(0.007)DG**S**(0.993)YIYPVENSNNIK	S	90
CD2717	*mldA*	Midcell localizing division protein	**S**(1)VVIPIK	S	573
CD0400	*etfB1*	Electron transfer flavoprotein β-subunit	ET(0.001)G**T**(0.999)LIR	T	24
CD0804	*etfB2*	Electron transfer flavoprotein β-subunit	ET(0.001)G**T**(0.999)LIR	T	24
CD1055	*etfB3*	Electron transfer flavoprotein β-subunit	LDPNT(0.005)G**T**(0.995)LIR	T	24
CD1324	*ftsK*	DNA FtsK/SpoIIIE translocase	ELQKA**T**(1)NENPVVDTKPEK	T	292
CD0395	*hadA*	Isocaprenoyl-CoA:2-hydroxyisocaproate CoA-transferase	VGQH**T**(1)VEVLK	T	374
CD0397	*hadB*	Subunit of O_2_-sensitive 2-hydroxy-isocaproyl-CoA dehydratase B	AFTNAQFE**T**(1)R	T	388
CD0880		Putative protease	KVDATVNV**T**(1)NQ	T	146
CD1125		Nitroreductase-family protein	VLNA**T**(1)KTGQNYSK	T	152
CD1768		Putative membrane protein	YNPEEDKA**T**(0.989)GT(0.011)LEK	T	186
Less abundant only in the Δ*prkC* mutant but not in the *CD2148* or double mutant					
CD1214	*spo0A*	Sporulation initiation regulator	GKVDTINQLFGY**T**(1)VHNTK	T	245
Less abundant only in the *CD2148* mutant but not in the Δ*prkC* or double mutant					
CD3484	*prfA*	Peptide chain release factor 1 (RF-1)	KLEVLEDTYKDL**S**(1)EK	S	16

Probabilities of localization of phosphosites are between parentheses.

### Proteins Involved in Metabolism Phosphorylated Under the Control of PrkC and CD2148

As observed in other firmicutes (12, 13, 19, 67), a phosphorylation dependent on STKs was observed for several key enzymes of metabolism (Fig. 6 and supplemental Fig. S7). Phosphorylations of PTS proteins, a P-sugar isomerase, and proteins involved in glycogen production or degradation were controlled by PrkC, CD2148, or both (supplemental Fig. S7 and supplemental Table S7). Contrary to other firmicutes (13, 19), enzymes of glycolysis were apparently not differentially phosphorylated in the mutants inactivated for the STKs in *C. difficile*. However, STK-dependent phosphorylations were identified for key enzymes of clostridial fermentation processes: a butyrate kinase, a phosphotransbutyrylase, two alcohol dehydrogenases but also the pyruvate–ferredoxin–oxidoreductase, two ferredoxins, and one copy of the pyruvate formate lyase (PflAB) (supplemental Fig. S7*A* and Table 1). CD2148 seems to be important for most of these phosphorylations (supplemental Fig. S7*A*). Interestingly, several of these proteins are also phosphorylated in *C. acetobutylicum* although the role of STKs in their phosphorylations remains to be established (21). Finally, several enzymes involved in the conversion of pantothenate to CoA were phosphorylated under the control of STKs (supplemental Fig. S7*C*).

*C. difficile* can use amino acids as energy source through Stickland reactions (68) that consist in the coupled fermentation of two amino acids in which one is oxidatively deaminated or decarboxylated and another (proline, glycine, or leucine) is reductively deaminated or reduced. Interestingly, several components of the leucine reductive pathway were found to be phosphorylated *in vivo* as observed in another phosphoproteomic analysis (22) (supplemental Fig. S7*B*). The phosphorylation of both HadA and EtfB on T143 was PrkC dependent, whereas HadB (T388) and EtfB (T24) phosphorylations required both the presence of PrkC and CD2148 (Table 1). These sites were more phosphorylated in the Δ*stp* mutant (Fig. 6). This suggests that STKs control this pathway that seems to be important for colonization (69).

### STK-Dependent Phosphorylations of Proteins Involved in Translation, Protein Folding, and Stress Responses

Several chaperones (GroES, GroEL, HtpG, and ClpX) were also phosphorylated under the control of STKs (supplemental Table S7). GroEL is also phosphorylated by an STK in *Bifidobacterium longum*, *Bacillus anthracis*, *B. subtilis*, *S. thermophilus*, and *M. tuberculosis* (17, 70). In *B. anthracis*, this phosphorylation facilitates the GroES–GroEL complex formation and controls biofilm formation. The ClpX and ClpC ATPases are phosphorylated in the phosphoproteome of *C. acetobutylicum*, whereas other Clp-associated ATPases are phosphorylated in *S. thermophilus* and *S. aureus* (17, 19). Since some key chaperones induced following a heat shock in *C. difficile* (49, 71) seem to be phosphorylated under the control of PrkC and/or CD2148, we tested the heat shock resistance of the WT strain and the Δ*prkC* or *CD2148* mutant. After exposure of the strains to 50 °C for 15 min, the survival of the Δ*prkC* mutant was five fold reduced compared with the WT strain (data not shown). This result suggested that PrkC plays a role in the heat shock response. However, the protein(s) targeted directly or indirectly by this PASTA-STK responsible for this phenotype remain(s) to be identified.

Three proteins (CD1634, CD1636, and CD1639) likely involved in tellurite resistance were also targets of phosphorylation under the control of STKs. We then tested the sensitivity of the different mutants to tellurite using a disk diffusion assay. We did not observe any difference in the growth inhibition area for the WT strain and the Δ*stp* mutant. By contrast, a significant increased sensitivity to tellurite was observed for the Δ*prkC*, *CD2148*, and double mutants (supplemental Fig. S8). These results suggested that tellurite resistance is controlled directly or indirectly by STKs. The phosphorylation of tellurite resistance proteins might contribute to this phenotype.

As observed in several firmicutes (17, 19, 21, 72) and detected in the phosphoproteome of *C. difficile* (supplemental Table S3 and (22)), several proteins involved in translation were phosphorylated under the control of STKs: six tRNA-synthetases, a 16S rRNA dimethyladenosine transferase, ribosomal proteins, the initiation factor, IF-2, and the EF-G and EF-Tu elongation factors (supplemental Table S7). The phosphorylation of EF-G and EF-Tu by the PrkC and/or YabT kinases in *B. subtilis* (4) likely plays a role during sporulation, germination, or outgrowth by modulating translation efficiency (73, 74). This might be also the case for the spore former *C. difficile*.

### PrkC and CD2148 Target Proteins Involved in Sporulation

**Fig. 7:**
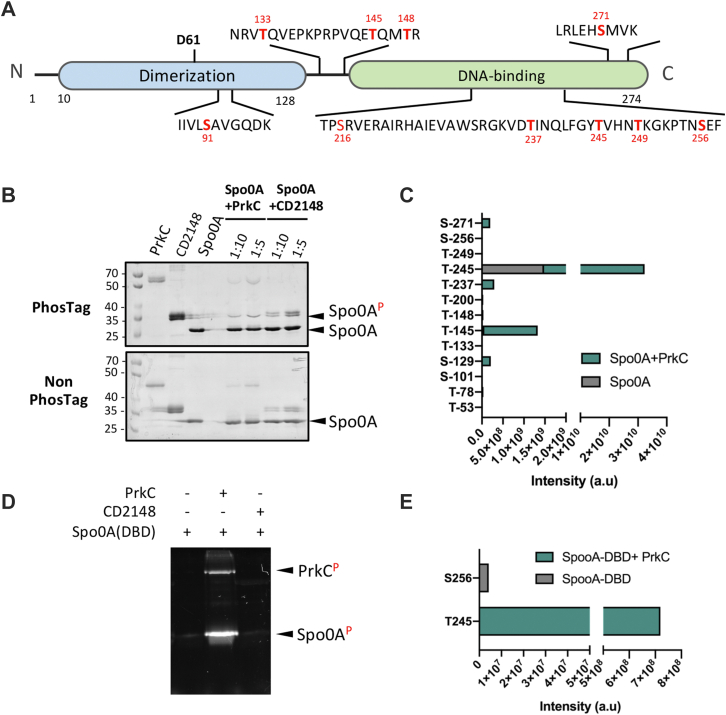
**Phosphorylation of Spo0A by PrkC.***A*, schematic presentation of the location of the different domains of Spo0A. The phosphorylated S and T residues identified *in vivo* are indicated in *red*. The D61 residue phosphorylated by the histidine kinases associated with Spo0A is also indicated. *B*, *in vitro* phosphorylation assay of Spo0A by the STKs at different ratio of kinase:substrate. Phosphorylation was visualized by a Phos-Tag acrylamide allowing the separation of phosphorylated and nonphosphorylated proteins. Molecular mass standards are shown on the *left*. *C*, mass spectrometry detection of Spo0A phosphorylation sites after *in vitro* phosphorylation reactions performed without or with PrkC. *D*, *in vitro* phosphorylation of the Spo0A-DBD by PrkC. Phosphorylation was visualized by Phos-Tag fluorescent gel stain reagents. *E*, mass spectrometry detection of Spo0A-DBD phosphorylation sites. DBD, DNA-binding domain; S, serine; STK, serine/threonine kinase; T, threonine.

Phosphorylation on S and T of proteins related to sporulation was detected. The sporulation protein YaaT (CD3548) was phosphorylated on S186 under the control of the two kinases and more phosphorylated in the *stp* mutant (Fig. 6 and Table 1). The anti–sigma factor antagonist SpoIIAA, which is known to be phosphorylated by the anti–sigma-factor SpoIIAB during sporulation in other bacteria (75), was found phosphorylated at S5, S56, S77, and S82 (supplemental Table S4) as also recently reported elsewhere (22). S56 is phosphorylated by SpoIIAB in other firmicutes (73), whereas S77 was found here to be phosphorylated under the control of PrkC (supplemental Fig. S5). Interestingly, the key sporulation factor Spo0A was also phosphorylated on multiple sites (Fig. 7*A*; supplemental Tables S4 and S10). One phosphosite (T245) exclusively absent in the Δ*prkC* mutant (Table 1) was shown to be more phosphorylated in the *Δstp* mutant (Fig. 6, *blue color*). Since *C. difficile* Spo0A plays a crucial role in sporulation initiation and controls virulence and metabolic adaptation (76), we wanted to confirm if Spo0A-His_6_ could be phosphorylated by PrkC *in vitro*. Purified full-length Spo0A was incubated with the catalytic domain of PrkC or CD2148 at ratio 1:10 and 1:5 in the presence of cold ATP. Using a Phos-Tag acrylamide gel to separate phosphorylated and nonphosphorylated proteins, we showed that PrkC was the only STK that efficiently phosphorylated Spo0A *in vitro* (Fig. 7*B*). We then analyzed the phosphorylation reactions by LC–MS/MS. Several residues were phosphorylated by PrkC-KD *in vitro*, most of them being detected as phosphorylated *in vivo* in a PrkC-dependent manner (T148, S271, T237, and T245) (Fig. 7, *A* and *C*). T245 was the residue with the highest level of phosphorylation detected *in vitro* in the presence of PrkC-KD in agreement with the *in vivo* results (Fig. 7*C*). Most of these phosphosites including T245 were located in the DNA-binding domain of Spo0A. Using the Phos-Tag fluorescent gel stain method, we confirmed that PrkC-KD but not CD2148-KD was able to phosphorylate the His_6_-tagged Spo0A-DNA-binding domain *in vitro* (Fig. 7*D*). Only two phosphorylated residues were detected by LC–MS/MS, S256 phosphorylated in a PrkC-independent manner and T245 specifically phosphorylated by PrkC (Fig. 7*E*). These results identified, for the first time, Spo0A as a substrate of the PASTA kinase, PrkC. However, the possible role of this phosphorylation in the control of sporulation and/or stationary phase processes remains to be established.

### Proteins Involved in Envelope Homeostasis and Protein Export Are Phosphorylated Under the Control of STKs

Several cell envelope–associated proteins were identified as possible substrates of PrkC and/or STP. In *C. difficile*, the two copies of the SecA ATPase, SecA1 and the accessory SecA2 proteins, were phosphorylated *in vivo* in the two phosphoproteomes (supplemental Table S3 and (22)). A phosphorylation of SecA is also detected in the phosphoproteome of *E. coli* and *M. tuberculosis* (77, 78), and the phosphorylation of SecA1 is dependent on Stk1/Stp in *S. aureus* (19). SecA1 and SecA2 were phosphorylated under the control of STKs (Table 1 and Fig. 6). It has been shown that the export of the S-layer protein (SlpA) and the cell wall protein (CwpV) is dependent on SecA2 (28). It is intriguing to note that these two proteins were also phosphorylated under the control of STKs (Fig. 6). SlpA is phosphorylated on its cell wall–binding domain, which interacts with the polysaccharide II and anchor SlpA (79). While CwpV confers phase variable phage resistance (80), SlpA may serve as phage receptor in *C. difficile* (81). Interestingly, we also detected two phage proteins (CD0905 and the duplicated CD2906/CD0958 proteins) that were phosphorylated under the control of PrkC and less phosphorylated in the Δ*stp* mutant (Fig. 6). CD2906 and CD0958 are interestingly related to SlpA in the string interaction network (Fig. 6). It is tempting to speculate the possible existence of a link between STKs, protein exportation, surface-associated proteins, and phage infection in *C. difficile*.

As observed in other firmicutes (6, 7, 82), proteins involved in cell wall homeostasis were identified as possible substrates of PrkC and/or STP. This included PG biosynthesis with the PrkC-dependent phosphorylation of GlmS, MurG adding *N*-acetylglucosamine to lipid, and MraY catalyzing the synthesis of lipid I (Fig. 6). It is worth noting that MraY is also phosphorylated in the phosphoproteome of *Helicobacter pylori* and *S. aureus* (83, 84), whereas Mur synthetases are targets of STKs in *M. tuberculosis*. PrkC-dependent phosphorylation of the endopeptidase CwlA controls its localization at the cell wall modulating cell separation as previously demonstrated (27). IreB was phosphorylated on T8 under the control of PrkC (Table 1) as observed in several firmicutes. IreB of *E. faecalis* negatively controls cephalosporin resistance (85). In *Listeria monocytogenes*, the phosphorylation of ReoM, the homolog of IreB, controls ClpCP-dependent proteolytic degradation of MurA involved in the first step of synthesis of PG precursors (86).

### Identification of Candidate Substrates of PrkC and Stp Involved in DNA Metabolism and Cell Division

Proteins involved in nucleotide synthesis (NrdD), DNA replication (DnaN), and recombination (RecX) were also candidate substrates for PrkC and STP (Table 1). The members of the RecX family of proteins have a unique capacity to regulate the catalytic activities of RecA, which is a protein phosphorylated by STKs in other firmicutes (87).

**Fig. 8:**
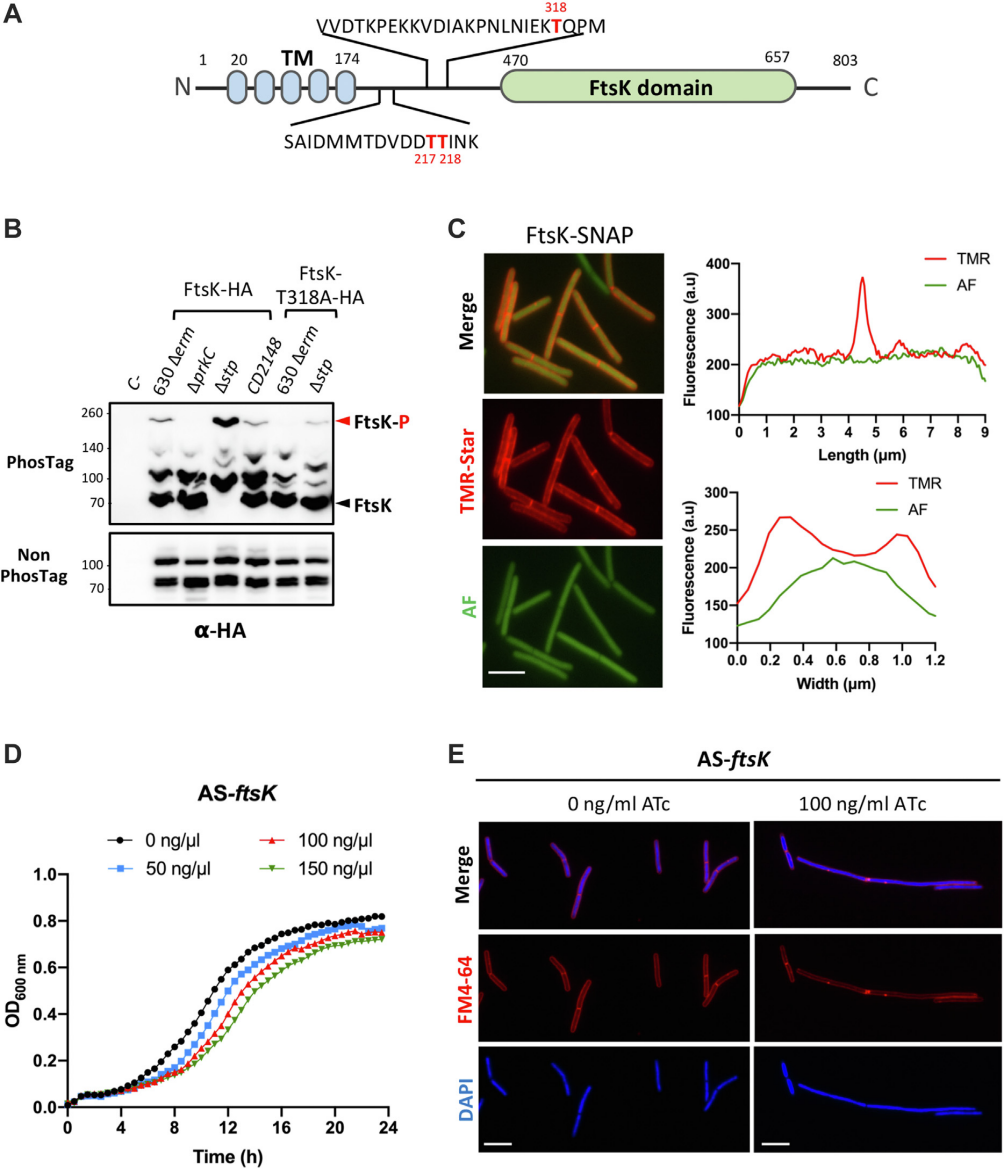
**PrkC-dependent phosphorylation of FtsK at residues localized within the flexible linker.***A*, schematic presentation of the different domains of FtsK. STK/STP-dependent phosphorylated residues detected *in vivo* are indicated in *red color* (supplemental Table S3). *B*, Phos-Tag and non–Phos-Tag SDS-PAGE with the whole-cell lysates of different strains expressing *ftsK*-HA or *ftsK*_T318A_-HA under the control of the P_*tet*_ promoter. The FtsK-HA protein was detected using an anti–HA-tag antibody. *C*, FtsK–SNAP localization. The expression of the P*_tet_ftsK*–SNAP fusion carried by a plasmid introduced into the WT strain was induced 2 h in the presence of 50 ng/ml of ATc. *Merge images*, TMR-Star fluorescent signal and autofluorescence (AF) are presented. Quantification of fluorescence in arbitrary units (a.u) along the major and minor axis for a typical cell. The scale bar represents 5 μm. *D*, growth curves of cells expressing an antisense RNA targeting the initiation of translation of the *ftsK* gene. *E*, fluorescence microscopy of cells expressing the *fts*K antisense (in the presence of 100 ng/ml of ATc) stained with the membrane dye FM4-64 and DAPI for the chromosome. The scale bar represents 5 μm. ATc, anhydrotetracycline; DAPI, 4′,6-diamidino-2-phenylindole; HA, hemagglutinin; STK, serine/threonine kinase; STP, serine/threonine phosphatase.

In firmicutes, PASTA-STKs play also a key role in the control of cell division and septum formation (7, 64, 88, 89). PrkC and STP are involved in the control of *C. difficile* morphogenesis (26, 27). We identified proteins of the divisome, such as SepF, DivIVA, and FtsZ, that were not phosphorylated in the Δ*prkC* and Δ*prkC CD2148* mutants and more phosphorylated in the Δ*stp* mutant (Fig. 6 and Table 1). MldA, a midcell localizing division protein, was not phosphorylated only in the double Δ*prkC CD2148* mutant and more phosphorylated in the Δ*stp* mutant (Fig. 6). In *S. pneumoniae*, phosphorylation of DivIVA by StkP negatively controls cell elongation and promotes cell division (88, 90). In *M. tuberculosis*, FtsZ is phosphorylated by PknA reducing its GTPase activity to possibly modulate Z-ring dynamics (91). Two additional proteins, WhiA and FtsK, which coordinate cell division with chromosome segregation, were also identified as candidate targets of PrkC and STP. Interestingly, the DNA translocase FtsK (CD1324) was phosphorylated on several residues (supplemental Tables S3 and S4). The phosphorylation of T217, T218, and T318 was abolished in the Δ*prkC* and Δ*prkC CD2148* mutants and increased in the Δ*stp* mutant (Fig. 6, Table 1 and supplemental Table S4). These phosphosites are located within the flexible linker (Fig. 8*A*) suggesting that phosphorylation could have an effect on the overall intrinsic flexibility of FtsK. While FtsK has been detected as phosphorylated in several phosphoproteomic studies (*C. acetobutylicum*, *M. bovis*, *M. smegmatis*, and *M. tuberculosis*), this protein has not been identified as a substrate of PASTA-STKs until now. To confirm its phosphorylation, we used strains expressing *ftsK*-HA under the control of a P*_tet_*-inducible promoter. Using Phos-Tag in Western blotting against anti-HA antibodies, we showed that FtsK-HA was phosphorylated in the WT and *CD2148* strains at similar levels but that this phosphorylation was abolished in the Δ*prkC* mutant. We also detected a higher level of phosphorylation in the Δ*stp* mutant in agreement with the phosphoproteomic data (Table 1 and Fig. 8*B*). After the replacement of T318 by the nonphosphorylatable alanine residue, we observed that the phosphorylation was undetectable in the WT background and strongly reduced in the Δ*stp* mutant (Fig. 8*B*). These results strongly suggest that T318 is phosphorylated under the control of PrkC. The residual phosphorylation detected in the Δ*stp* mutant is probably because of the additional phosphosites (T217 and T218) (Table 1). Localization of FtsK was then determined using a P_*tet*_*–*SNAP–FtsK fusion. After induction, we detected the SNAP–FtsK protein around the membrane with an enrichment at midcell (Fig. 8*C*). FtsK colocalized with PrkC at the cell septum even if its localization pattern is slightly different from that of PrkC. To further confirm that FtsK plays a role in cell division, we used a knockdown strategy by using an antisense RNA expressed under the control of a P_*tet*_-inducible promoter that targets the 5′ end region of the *ftsK* mRNA and sequesters the ribosomal-binding site within an RNA duplex. This strategy has been successfully used for the depletion of essential genes in *C. difficile* (28, 92, 93). Upon induction of AS-*ftsK* with increasing concentration of ATc, we observed not only a growth defect but also an elongation of the cells, a phenotype also detected for the Δ*prkC* mutant (Fig. 8, *D* and *E*).

## Discussion

**Fig. 9:**
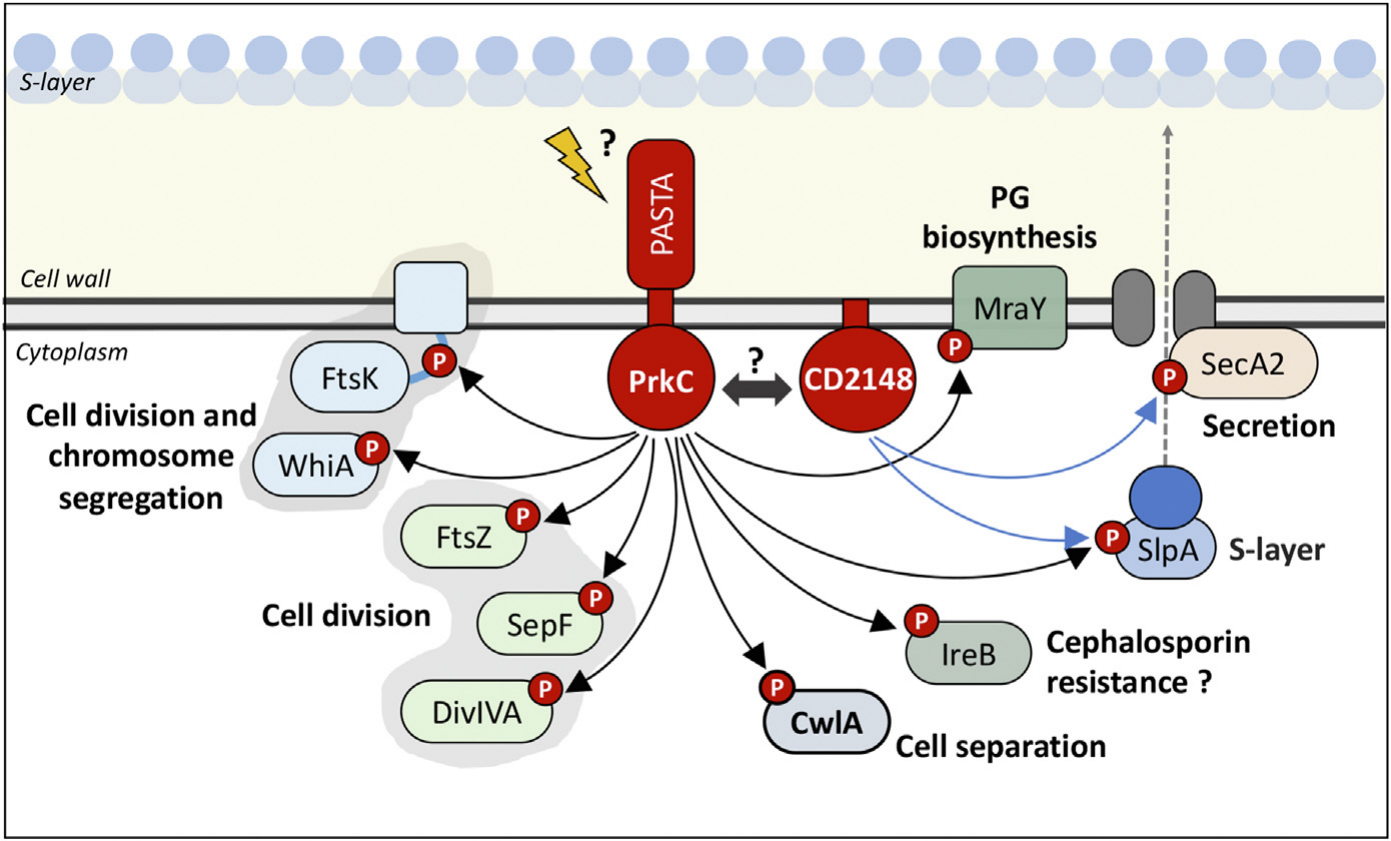
**Major targets of *Clostridioides difficile* STKs involved in cell division and envelope homeostasis.** Schematic representation of major phosphorylated proteins involved in cell division and envelope homeostasis identified as STK/STP dependent in this study. *Black and blue arrows* mean PrkC- and CD2148-dependent phosphorylation, respectively. PrkC could sense extracellular signals generated during PG synthesis (muropeptides, lipid II, or other) through its PASTA domains. PASTA, penicillin-binding and STK-associated; PG, peptidoglycan; STK, serine/threonine kinase; STP, serine/threonine phosphatase.

The large-scale comparative phosphoproteome analysis of *C. difficile* that we have presented here provides new knowledge regarding the specific proteins, pathways, and cellular processes that may be regulated by STK-dependent phosphorylation in this human enteropathogen. By this approach, we were able to identify candidate substrates for PrkC and CD2148, and for the phosphatase, STP. Specifically, we have provided strong evidence for PrkC-dependent phosphorylation of FtsK and Spo0A. However, given the descriptive nature of phosphoproteomic and the inability to distinguish between direct and indirect effects of STKs, targeted approaches will be necessary to confirm substrates directly phosphorylated by each STK or some that can depend on both kinases. Our results lead to several questions regarding the role of these kinases in *C. difficile* physiology. The phosphoproteomic data suggest a broader role for PrkC with a more limited and partially overlapping role for CD2148. Both kinases target directly or indirectly pathways required for metabolism, translation, protein folding, and stress response. By contrast, proteins involved in cell division and PG metabolism (Fig. 9) seem to be mainly phosphorylated under the control of the PASTA kinase, PrkC, as observed in most firmicutes (6). The phosphorylation under the control of the PASTA kinase of different components involved in the synthesis or degradation of PG could contribute to several phenotypes of the *C. difficile* Δ*prkC* mutant including the decreased amount of PG detected and the reduced resistance to several antimicrobial compounds targeting the envelope (26, 27). With the exception of CwlA, the precise molecular mechanisms involving these proteins and PrkC to control envelope homeostasis remain to be deciphered. As observed in *S. pneumoniae*, *M. tuberculosis*, *S. aureus*, *Corynebacterium glutamicum*, and *Streptococcus coelicolor*, the PASTA-STK of *C. difficile*, PrkC, controls directly or indirectly the phosphorylation of several proteins involved in septum formation and cell division (Fig. 9). This is reminiscent with the defect of cell morphology and septation observed for the *C. difficile* Δ*prkC* mutant (26). Whereas DivIVA and FtsZ have been identified as STK substrates before in other organisms, PASTA kinase–dependent phosphorylation of SepF and FtsK is less well described. The precise role of these phosphorylations in the control of the cell division process or its coupling with PG synthesis in *C. difficile* remains to be deciphered. We cannot exclude that a cell wall stress, which could be detected by the extracellular PASTA domains, could allow to identify additional PrkC targets in cell division and PG biosynthesis machinery as shown recently for *E. faecalis* and *L. monocytogenes* (94, 95). It is also interesting to note that we do not identify two-component systems as targets of PrkC in *C. difficile* contrary to the data obtained in *B. subtilis*, *E faecalis*, and *S. aureus* (19, 94, 96). This is in agreement with the low impact of STK inactivation on global proteome even if one regulator of the Crp family and a diguanylate kinase are identified as candidate substrates of PrkC. Several targets are phosphorylated specifically under the control of CD2148 *in vivo*, but the role of this atypical STK is less clear. CD2148 lacks six of the 12 conserved residues commonly found in STKs (supplemental Fig. S2, *red asterisks*) including the D and N residues (replaced by S134 and S139), which are important for stability of Mg^2+^ ion binding and catalytic activity (97). Its phosphorylation *in vitro* under the conditions tested depends on PrkC, and direct evidence for the phosphorylation of candidate targets *in vitro* by CD2148 is still lacking. The absence of detection of CD2148 phosphorylation *in vivo* even in the presence of PrkC might be because of either an absence of known signals for this kinase triggering its activation or an efficient transfer to its substrates. We also cannot completely exclude that CD2148 may be a pseudokinase (98) despite the fact that the number of substrates phosphorylated under its control in our phosphoproteome is high. Finally, the *in vivo* and *in vitro* probable interplay between the two STKs, and the possible role of PrkC in CD2148-dependent phosphorylation remains to be investigated (Fig. 9).

Strikingly, we observe a significant number of proteins phosphorylated on S/T in a PrkC- and CD2148-independent manner in *C. difficile* 630Δ*erm* strain. This suggests the existence of other non–Hanks-kinase pathways for phosphorylation. In firmicutes, RsbW, SpoIIAB, and the HPr-kinase/phosphorylase are specialized kinases for RsbV, SpoIIAA, and HPr, respectively (51, 52). Compared with *B. subtilis*, the HPr-kinase seems to play a less important role in the control of carbon catabolite repression in *C. difficile* (52), and we cannot exclude that this kinase may have other substrates than HPr. Finally, additional uncharacterized S/T-kinases could be present in *C. difficile* as recently found in *E. coli* (99). Similarly, the strong enrichment for phosphosites corresponding to T residues among the candidate substrates common to PrkC and STP suggests the possible existence of other phosphatase(s) more specific for pS and maybe also for CD2148-dependent targets in *C. difficile*. SpoIIE, which is involved as a phosphatase of some substrates of the STK YabT in *B. subtilis* (87) or CD2272, a putative serine phosphatase, might be good candidates.

STKs are considered as key integration nodes in signaling pathways. Our optimized protocol allows the identification of an important number of phosphorylation events in agreement with another recent phosphoproteomic study (22) and more importantly of candidate substrates for the two Hanks-kinases in *C. difficile*. Our results provide new insights into the multiple levels of control allowing adaptive responses of this enteropathogen in the complex gut environment and pave the way to the development of new therapeutic strategies targeting phosphoregulated pathways.

## Data Availability

The MS proteomics data have been deposited to the ProteomeXchange Consortium (http://proteomecentral.proteomexchange.org) *via* the PRIDE partner repository with the dataset identifier PXD029827 (100). The list of phosphopeptides and the spectra for the optimization step can be viewed on this link: https://msviewer.ucsf.edu/prospector/cgi-bin/mssearch.cgi?report_title=MS-Viewer&search_key=bymwsuikwx&search_name=msviewer. The global phosphoproteome data from the analysis of the different strains have also been loaded into MSviewer at this link: https://msviewer.ucsf.edu/prospector/cgi-bin/mssearch.cgi?report_title=MS-Viewer&search_key=lhik1ga8x8&search_name=msviewer. Spo0A phosphosites described in the article can be viewed on this link: https://msviewer.ucsf.edu/prospector/cgi-bin/mssearch.cgi?report_title=MS-Viewer&search_key=ftbg0udqzm&search_name=msviewer. PrkC and CD2148 phosphosites can be viewed on this link: https://msviewer.ucsf.edu/prospector/cgi-bin/mssearch.cgi?report_title=MS-Viewer&search_key=9gvyuyvfcv&search_name=msviewer.

## Supplemental data

This article contains supplemental data.

## Conflict of interest

The authors declare no competing interests.
