# Supplementary material for: In-Depth Characterization of the Clostridioides difficile Phosphoproteome to Identify Ser/Thr Kinase Substrates
Source: Mol Cell Proteomics. 2022 Oct 14;21(11):100428. doi: 10.1016/j.mcpro.2022.100428 (PMC9674922; doi:10.1016/j.mcpro.2022.100428)
Supplement: Supplemental Figures S1–S8 [file mmc1.pdf]

A

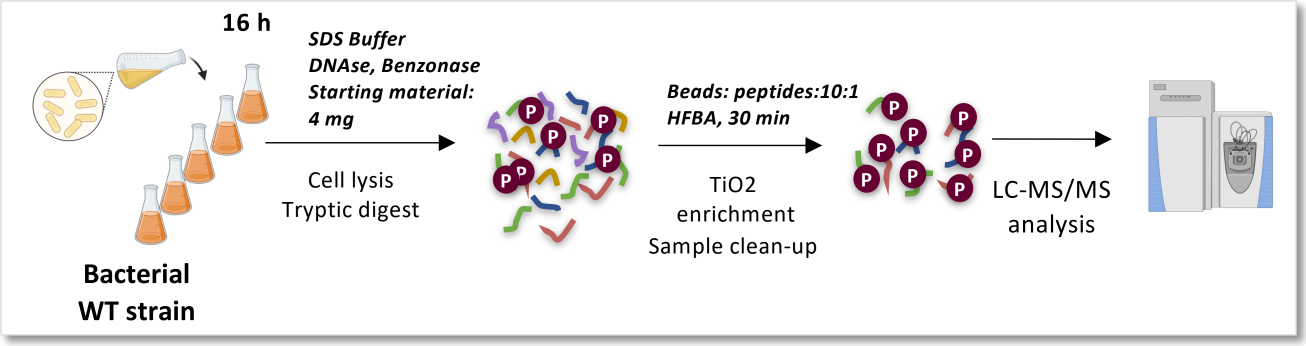

B

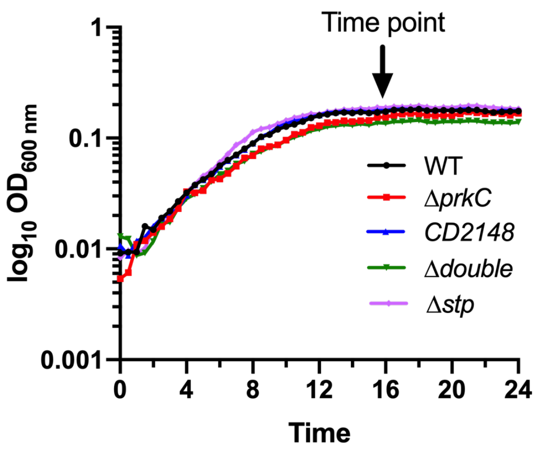

C

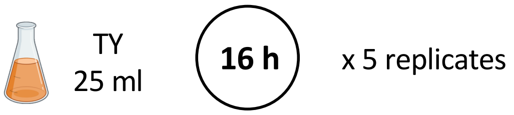

Strain	OD_{600} nm	Total protein amount (mg)
WT	1.85 ± 0.09	15.4 ± 0.74
ΔprkC	1.70 ± 0.09	15.6 ± 1.84
CD2148	1.80 ± 0.07	14.3 ± 0.89
Δdouble	1.62 ± 0.08	12.6 ± 1.42
Δstp	1.88 ± 0.04	14.7 ± 0.94

Figure S1. A, Optimized protocol for S/T/Y phosphopeptides enrichment in *C. difficile.* **B**, Growth curves of *C. difficile* strains in TY medium in a 24. **C**, Optical density at 600 nm and total protein amount (mg) extracted from *C. difficile* strains cultured in TY for 16 h.

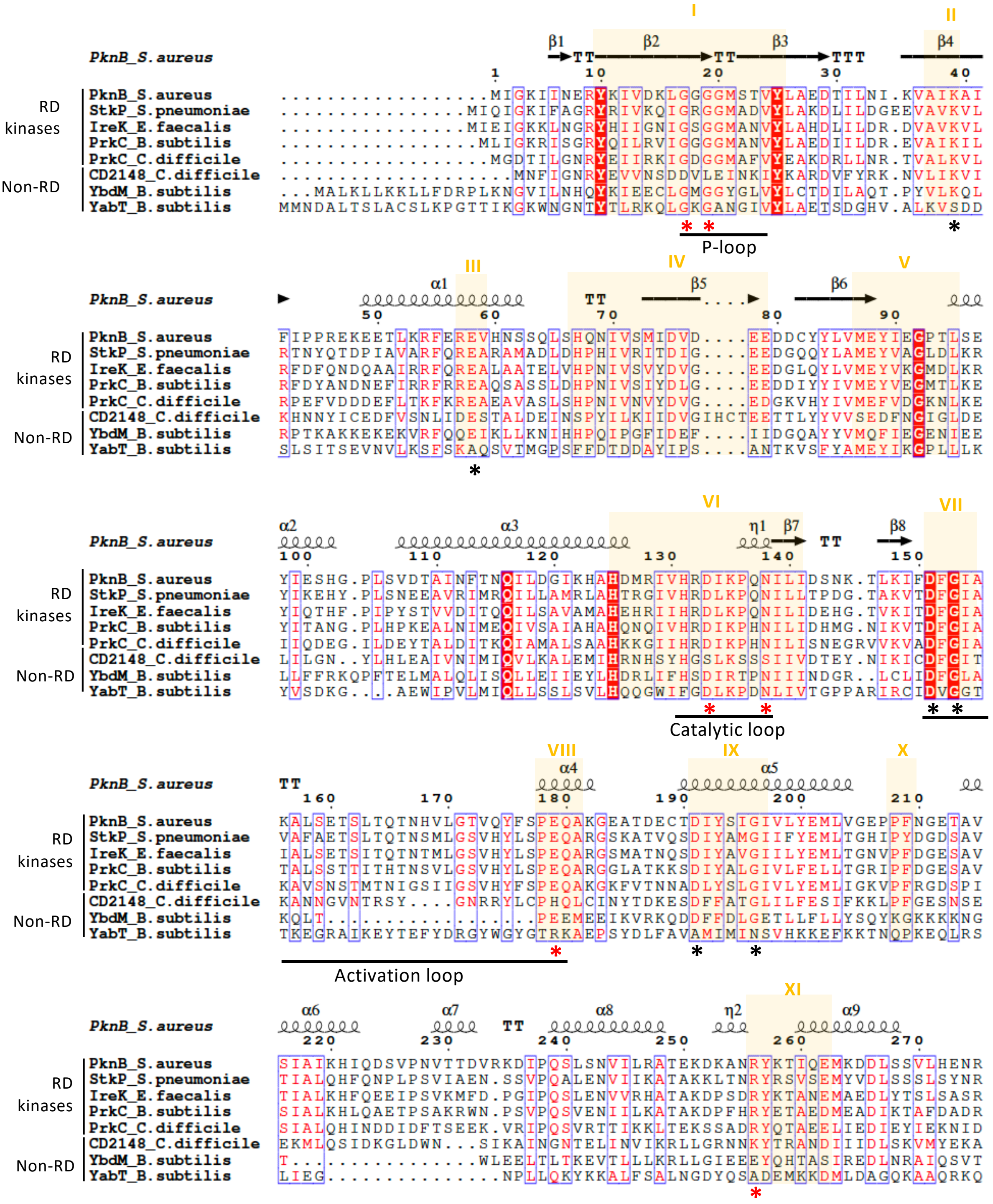

Figure S2. Amino acid sequence alignment of kinase domain of PrkC and CD2148 with bacterial STKs. PrkC and CD2148 were aligned to the kinase domain of bacterial STKs using ESPript. PknB (*Staphylococcus aureus);* StkP (*Streptococcus pneumoniae);* IreK *(Enterococcus faecalis);* PrkC, YbdM, YabT *(Bacillus subtilis)*. Conserved residues are shaded in red. Hanks-type conserved motifs are indicated on top of the sequence as roman numbers I-XI, the twelve invariant residues are denoted by asterisks (red asterisks: non conserved in CD2148). Functionally important motifs are indicated below the sequence. Secondary structure elements from PknB (PDB code: 4EQM) are presented on the top.

A

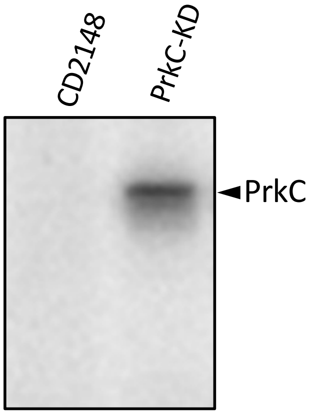

B

PrkC	Phosphoresidue	LP
Activation loop	S-160	0.56
	S-162	0.75
	T-163	0.98
	T-165	0,91
Helical lobe	S-214	1
	S-217	0.88
Juxtamembrane	T-287	1
	T-302	1
Other	S-35	1
	T-243	0,96

C

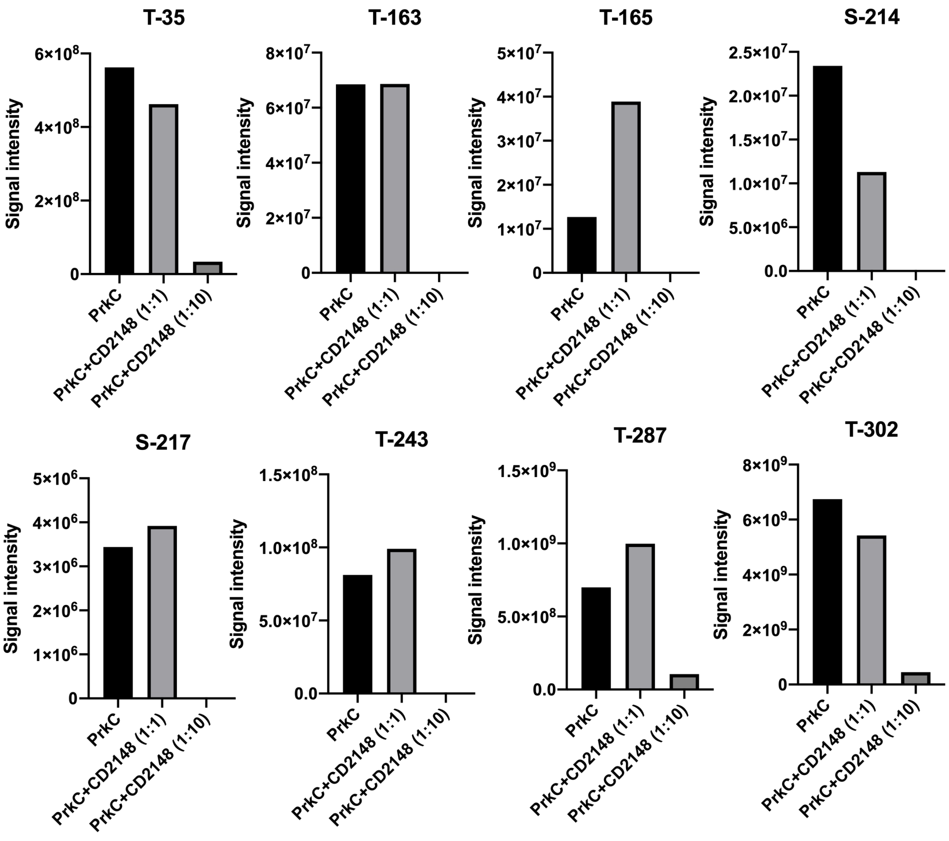

Fig S3. A, Phosphorylation *in vitro* of the complete CD2148 protein or PrkC-KD (used as a control) in the presence of P^{32}-ATP (X mM). **B**, PrkC phosphorylation residues confirmed *in vitro* by LC-MS/MS. LP is the localization probability of the identified phosphosite. **C**, Signal intensities of PrkC-KD phosphorylation residues in presence or absence of CD2148-KD detected by LC-MS/MS with LP>0.75.

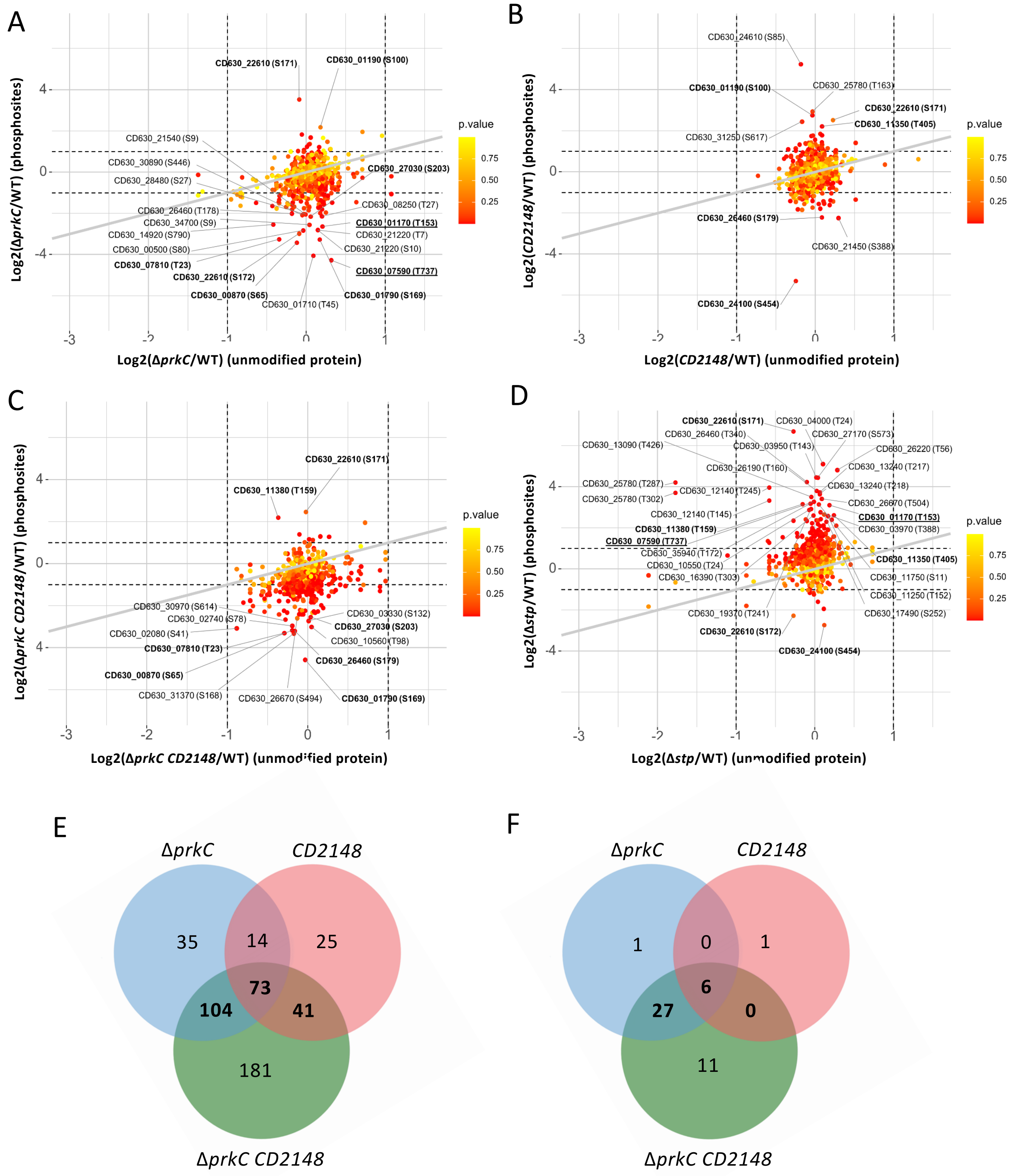

Figure S4. Log2 fold-changes between intensities quantified in Δ*prkC* (A), *CD2148* (B), the double mutant Δ*prkC CD2148* (C), Δ*stp* (D) and the WT strain for each phosphopeptide when compared to the ones quantified for their unmodified protein. Red gradients are related to the p-values of testing if both log2 fold-changes are equal (i.e. log2(FC phosphopeptide)=log2(FC protein)). The names of the phosphosites that evolve the most differently from their corresponding protein are shown in the graphs: they are in bold if they are among the most varying in two or more comparisons. E and F) Venn diagram showing overlap of the phosphoproteins detected for each strain. Proteins significantly less phosphorylated in the kinase mutants (Δ*prkC*, *CD2148* or double) compared to WT strain (E) or also more phosphorylated in the Δ*stp* mutant compared to WT strain (F) are indicated (Table S5 and S7).

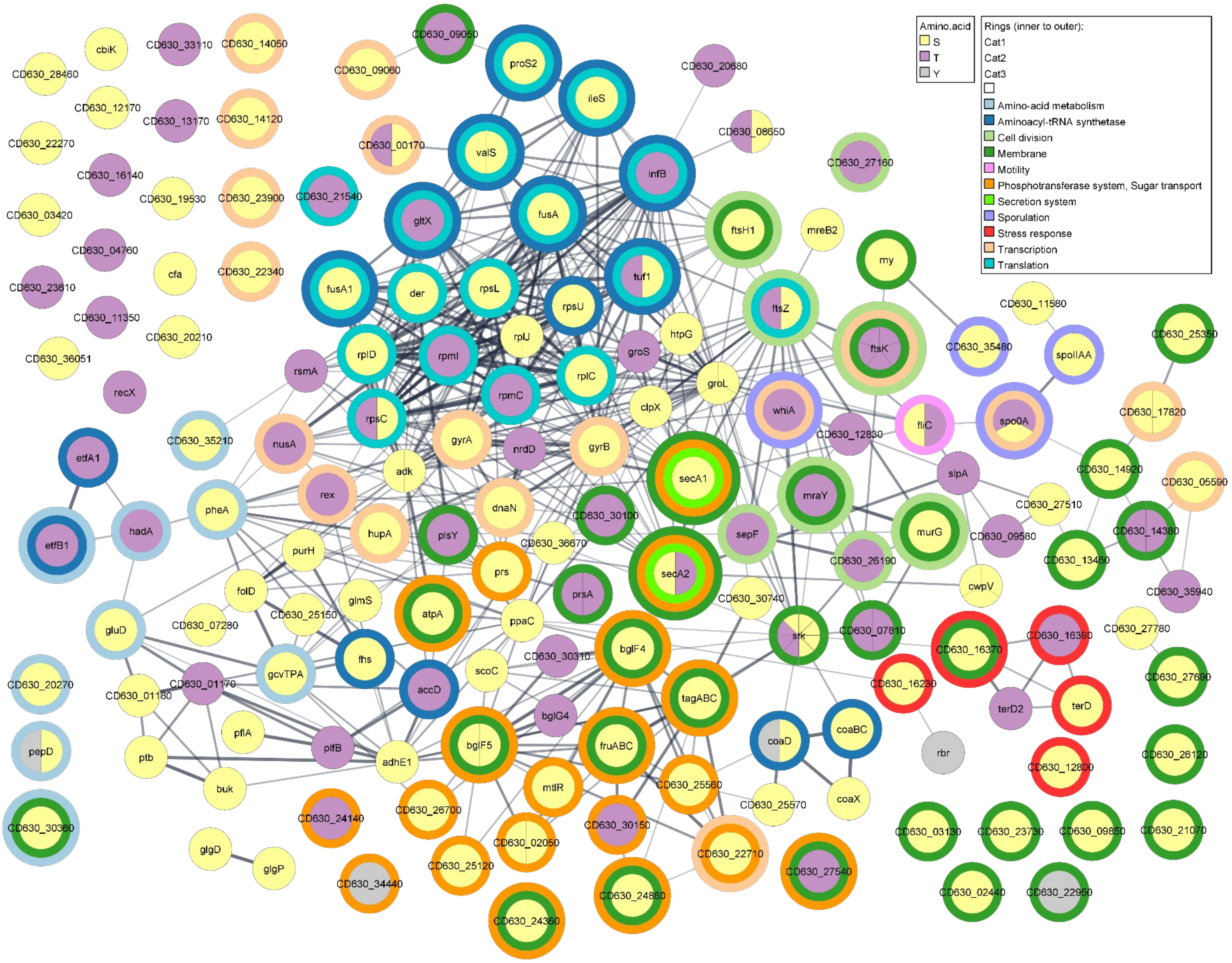

Figure S5. **Functional analysis of protein-protein interaction for proteins that are probable PrkC targets**. 177 proteins (200 phosphopeptides) were identified with phosphopeptides less abundant in the Δ*prkC* and the Δ*prkC CD2148* mutants compared to WT.

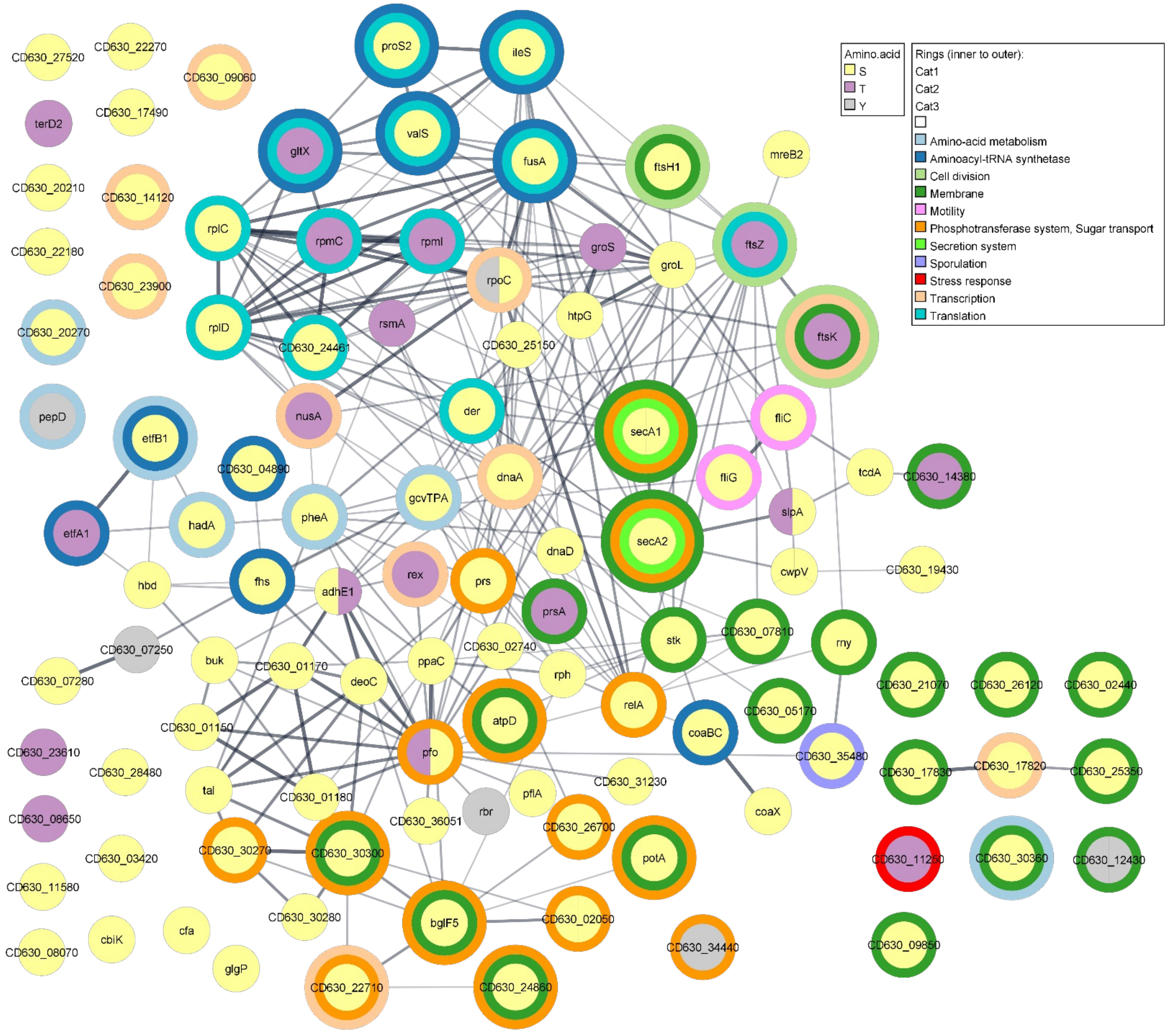

Figure S6. Functional analysis of protein-protein interaction for proteins that are probable CD2148 targets. 114 proteins (120 phosphopeptides) were identified with phosphopeptides less abundant in the *CD2148* and Δ*prkC CD2148* mutants compared to WT.

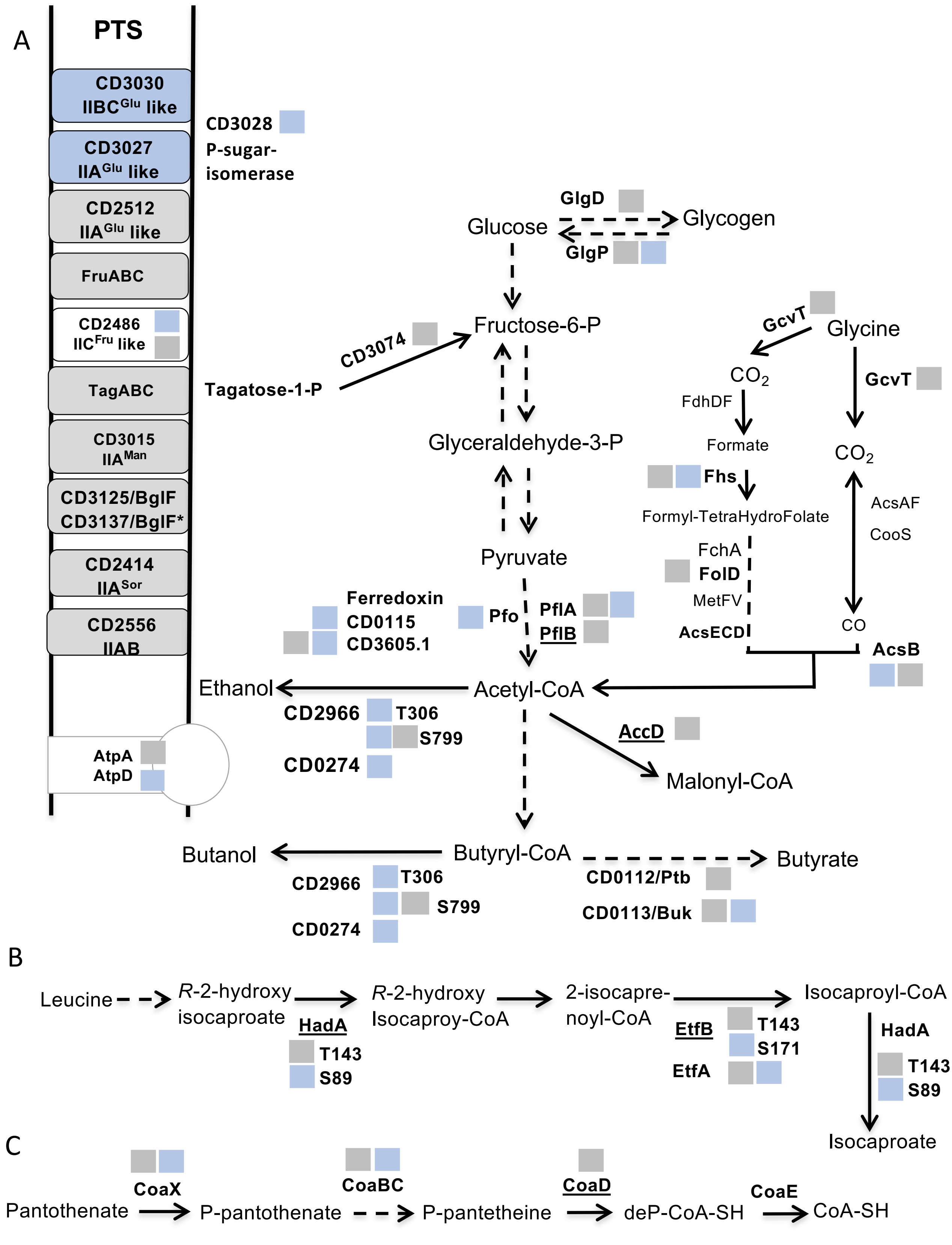

Figure S7. Metabolic enzymes phosphorylated *in vivo* under the control of the STK kinases. Phosphoproteins involved in metabolic pathways based on the KEGG (Kyoto Encyclopedia of Genes and Genomes), GeneBank and Uniprot database, including (**A**) the central carbon metabolism (sugar uptake, glycolysis, fermentation pathways), (**B**) the leucine catabolism and (**C**) CoA-SH synthesis. The gray and blue squares (or the colored forms for transporters) represent proteins identified as phosphorylated under the control of PrkC (phosphosites absent or less phosphorylated in both Δ*prkC* mutant and Δ*prkC CD2148* mutant compared to the WT strain) or CD2148 (phosphosites absent or less phosphorylated in both CD2148 mutant andΔ*prkC CD2148* mutant compared to the WT strain), respectively . When a phosphosite is absent or less phosphorylated in all three mutants, both a gray and a blue squares are indicated. When two different sites are phosphorylated for a protein with a different STK-dependent profile, the number of the S or T phosphorylated residue is indicated. The proteins with a phosphosite more abundant in the Δ*stp* mutant are overlined. The data were obtained from supplementary Table 7.

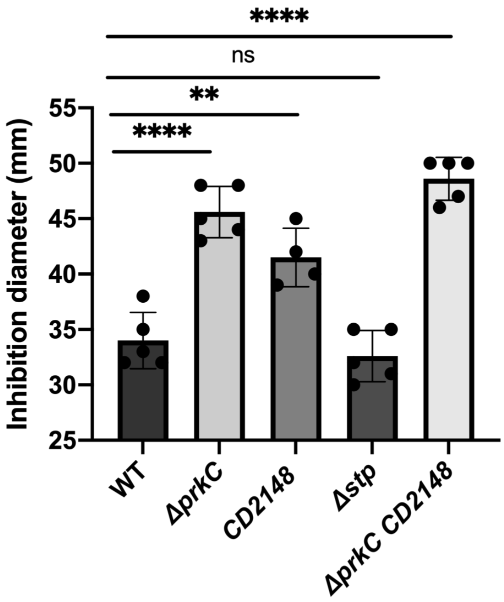

Figure S8. **Sensitivity to tellurite.** Histograms representing the diameters of growth inhibition area after 24 h incubation on BHI plates for WT, Δ*prkC, CD2148,* Δ*stp* and Δ*prkC CD2148* mutants with 200 mM tellurite. Data were analyzed by unpaired *t* test. **** $P < 0.0001$; ** $P = 0.0011$ and ns = 0.4676. Average values and standard error of the means are calculated from 5 independent experiments.
