# Supplementary material for: In-Depth Characterization of the Clostridioides difficile Phosphoproteome to Identify Ser/Thr Kinase Substrates
Source: Mol Cell Proteomics. 2022 Oct 14;21(11):100428. doi: 10.1016/j.mcpro.2022.100428 (PMC9674922; doi:10.1016/j.mcpro.2022.100428)
Supplement: Supplemental Tables S1 and S2 [file mmc2.docx]

**Supplementary Table 1.** Strains and plasmids used in this study

|  | ***E. coli* strains** |  |
| --- | --- | --- |
| NEB |  |  |
| HB101 (RP4) | *supE*44 *aa*14 *galK*2 *lacY*1 ∆ (*gpt-proA*) 62 *rpsL*20 (Str^R^)*xyl-5 mtl-1 recA*13 ∆ (*mcrC-mrr*) *hsdS*_B_(r_B_^-^m_B_) RP4 (Tra^+^ IncP Ap^R^ Km^R^ Tc^R^) | Laboratory stock |
| BL21 | F^−^ompT gal dcm lon hsdS_B_(r_B_^-^ m_B_^-^) λ(DE3 [lacI lacUV5-T7 gene 1 ind1 sam7 nin5]) | Novagen |
|  | ***C. difficile* strains** |  |
| 630∆*erm* | wild-type | Laboratory stock |
| CDIP631 | 630∆*erm* ∆*prkC* | Cuenot *et al.,* 2019 |
| CDIP823 | 630∆*erm* ∆*stp* | Garcia-Garcia *et al.,* 2021 |
| CDIP736 | 630∆*erm CD2148::erm* | Garcia-Garcia *et al.,* 2021 |
| CDIP738 | 630∆*erm* ∆*prkC CD2148::erm* | Garcia-Garcia *et al.,* 2021 |
| CDIP219 | 630∆*erm* pDIA6103 | Laboratory stock |
| CDIP1357 | 630∆*erm* P_tet_ SNAP*^Cd^-prkC* | Cuenot *et al.,* 2019 |
| CDIP1432 | 630∆*erm* P_tet_ SNAP*^Cd^-prkC* ∆SGN | This work |
| CDIP1433 | 630∆*erm* P_tet_ SNAP*^Cd^-prkC* ∆PASTA-∆SGN | This work |
| CDIP1467 | 630∆*erm* P_tet_ *ftsK*-SNAP*^Cd^* | This work |
| CDIP1478 | 630∆*erm* P_tet_ *ftsK*-HA | This work |
| CDIP1479 | 630∆*erm* ∆*prkC* P_tet_ *ftsK*-HA | This work |
| CDIP1480 | 630∆*erm CD2148::erm* P_tet_ *ftsK*-HA | This work |
| CDIP1481 | 630∆*erm* ∆*stp* P_tet_ *ftsK*-HA | This work |
| CDIP1553 | 630∆*erm* P_tet_ *ftsK* T318A-HA | This work |
| CDIP1555 | 630∆*erm* ∆*stp* P_tet_ *ftsK* T318A-HA | This work |
| CDIP1617 | 630∆*erm* P_tet_ AS *ftsK* | This work |
|  | **Plasmids** |  |
| pDIA6103 | pRPF185 Δ*gusA* | Soutourina *et al*., 2013 |
| pDIA6855 | pDIA6103-P_tet_-SNAP^Cd^-*prkC* | Cuenot *et al.,* 2019 |
| pDIA6917 | pDIA6103-P_tet_-SNAP^Cd^-*prkC* ∆SGN | This work |
| pDIA6918 | pDIA6103-P_tet_-SNAP^Cd^-*prkC* ∆PASTA ∆SGN | This work |
| pDIA6951 | pDIA6103-P_tet_ *ftsK-* SNAP^Cd^ | This work |
| pDIA6908 | pDIA6103-P_tet_ *ftsK* | This work |
| pDIA6946 | pDIA6103-P_tet_ *ftsK*-HA | This work |
| pDIA7010 | pDIA6103-P_tet_ *ftsK* T318A-HA (phospho-ablative) | This work |
| pDIA7044 | pDIA6103-P_tet_ AS *ftsK* | This work |
| pDIA6406 | pQE30-*prkC-*KD | Garcia-Garcia *et al.,* 2021 |
| pDIA6407 | pQE30-*CD2148-*KD | Garcia-Garcia *et al.,* 2021 |
| pDIA7208 | pQE30-*CD2148* | This work |
| pWKS1245 | pET21b-*spo0A*-DBD-His_6_ | Rosenbusch *et al*, 2012 |
| pWKS1251 | pET21b-*spo0A*-His_6_ | Rosenbusch *et al*, 2012 |

- Cuenot E, Garcia-Garcia T, Douche T, Gorgette O, Courtin P, Denis-Quanquin S, Hoys S, Tremblay Y, Matondo M, Chapot-Chartier MP, Janoir C, Dupuy B, Candela T, Martin-Verstraete I. 2019. The Ser/Thr Kinase PrkC Participates in Cell Wall Homeostasis and Antimicrobial Resistance in *Clostridium difficile*. *Infection and immunity*, *87*(8), e00005-19.
- Soutourina OA, Monot M, Boudry P, Saujet L, Pichon C, Sismeiro O, Semenova E, Severinov K, Le Bouguenec C, Coppee JY, Dupuy B, Martin-Verstraete I. 2013. Genome-wide identification of regulatory RNAs in the human pathogen *Clostridium difficile*. PLoS Genet 9:e1003493
- Garcia-Garcia, T., Poncet, S., Cuenot, E., Douché, T., Gianetto, Q. G., Peltier, J., Courtin, P., Chapot-Chartier, M.-P., Matondo, M., Dupuy, B., Candela, T., and Martin-Verstraete, I. (2021) Ser/Thr Kinase-Dependent Phosphorylation of the Peptidoglycan Hydrolase CwlA Controls Its Export and Modulates Cell Division in *Clostridioides difficile*. *mBio* May 18;12(3):e00519-21.
- Rosenbusch K. E, Bakker D. , Kuijper E.J. and Smits W. K.. 2012. *C. difficile* 630∆*erm* Spo0A Regulates Sporulation, but Does Not  Contribute to Toxin Production, by Direct High-Affinity Binding to  Target DNA. PloS One. 7(10):e48608

**Supplementary Table 2.** Oligonucleotides used in this study

| **Primers** | **Sequence (5’→3’)** | **Features** |
| --- | --- | --- |
| IMV988 | AGGCCTGGAGCTCAGATCTG | GA-pDIA6103 |
| IMV989 | GGATCCTATAAGTTTTAATAAAACTTTAA | GA-pDIA6103 |
| TG33 | CAGATCTGAGCTCCAGGCCTCTGTGAAGGGGTAAAACTTGCTAA | GA-pDIA6103 FtsK Fwd |
| TG34 | TTAAAGTTTTATTAAAACTTATAGGATCCAGTGCTTTTTCTATTTTTATAACTGACA | GA-pDIA6103 FtsK Rev |
| TG35 | TATTAAGCGTAATCTGGAACATCATATGGATATTCACCTTCTAAATTCTGCAA | FtsK-HA Cter Rev |
| TG36 | TTGTCAGTTATAAAAATAGAAAAAGC | FtsK-HA Cter Fwd |
| TG67 | TTCACCTTCTAAATTCTGCAAA | pDIA6103-FtsK SNAP Rev |
| TG68 | TGTCAGTTATAAAAATAGAAAAAGCA | pDIA6103-FtsK SNAP Fwd |
| TG69 | TTTGCAGAATTTAGAAGGTGAAGGATCCGCAGCTGCTGATAAAGATTGTGAAATGAAGAGAACC | GA-SNAP Fwd |
| TG70 | TGCTTTTTCTATTTTTATAACTGACATTACCCAAGTCCTGGTTTCCCCA | GA-SNAP Rev |
| TG71 | GCATCTAAAGACAGCAATTTAAC | FtsK int 1 |
| TG72 | GTCCAGGAGTCAAGGTAAGTAA | FtsK int 2 |
| TG88 | ATTGTAGCAGAGCCAGTTAATGAG | ftsK T318A Fwd |
| TG89 | ACTCATAGGTTGAGCTTTTTCTATATT | ftsK T318A Rev |
| IMV1190 | GGGGATCCTGTGAAGGGGTAAAACTTGCT | AS ftsK 5’ BamHI, |
| IMV1191 | GAAGGCCTAACACAGGTATCCATCCCATA | AS ftsK 3’ StuI |
| IMV1118 | GGGATTTCTCACATAAAATAGAGTTATGTTGGAGTCTGTGTATTTGA | SNAP PrkC ∆SGN |
| IMV1120 | GGGATTTCTCACATAAAATAGAGTTATCCAGCAAATAAAAACTTATATGC | SNAP PrkC ∆ext |
